# Dissecting the Genetic and Etiological Causes of Primary Microcephaly

**DOI:** 10.3389/fneur.2020.570830

**Published:** 2020-10-15

**Authors:** Francesca Jean, Amanda Stuart, Maja Tarailo-Graovac

**Affiliations:** ^1^Department of Biochemistry and Molecular Biology, Cumming School of Medicine, University of Calgary, Calgary, AB, Canada; ^2^Department of Medical Genetics, Cumming School of Medicine, University of Calgary, Calgary, AB, Canada; ^3^Alberta Children's Hospital Research Institute, University of Calgary, Calgary, AB, Canada

**Keywords:** microcephaly, neurogenesis, genetics, cell cycle, rare disease (RD)

## Abstract

Autosomal recessive primary microcephaly (MCPH; “small head syndrome”) is a rare, heterogeneous disease arising from the decreased production of neurons during brain development. As of August 2020, the Online Mendelian Inheritance in Man (OMIM) database lists 25 genes (involved in molecular processes such as centriole biogenesis, microtubule dynamics, spindle positioning, DNA repair, transcriptional regulation, Wnt signaling, and cell cycle checkpoints) that are implicated in causing MCPH. Many of these 25 genes were only discovered in the last 10 years following advances in exome and genome sequencing that have improved our ability to identify disease-causing variants. Despite these advances, many patients still lack a genetic diagnosis. This demonstrates a need to understand in greater detail the molecular mechanisms and genetics underlying MCPH. Here, we briefly review the molecular functions of each MCPH gene and how their loss disrupts the neurogenesis program, ultimately demonstrating that microcephaly arises from cell cycle dysregulation. We also explore the current issues in the genetic basis and clinical presentation of MCPH as additional avenues of improving gene/variant prioritization. Ultimately, we illustrate that the detailed exploration of the etiology and inheritance of MCPH improves the predictive power in identifying previously unknown MCPH candidates and diagnosing microcephalic patients.

## Introduction

Autosomal recessive primary microcephaly (MCPH) is a rare, heterogeneous disorder characterized by an occipitofrontal diameter >2 or 3 standard deviations below the mean at birth, after accounting for sex and ethnicity. MCPH patients typically have a simplified cerebral cortical gyral pattern (lissencephaly) although overall brain architecture is generally normal. MCPH is also frequently associated with other clinical features such as intellectual impairment, short stature, and mild seizures—since these features frequently overlap with other syndromes, it is likely that MCPH is part of a disease spectrum. The rate of incidence varies between 1 in 10,000, in populations where consanguineous marriages are common, and 1 in 250,000 in non-consanguineous populations (1, 2). Although there are some examples of dominantly inherited primary microcephaly, it is typically inherited in an autosomal recessive manner.

There are currently 25 MCPH-associated genes listed in the Online Mendelian Inheritance in Man (OMIM) database (accessed as of April 2020): *MCPH1, WDR62, CDK5RAP2, KNL1, ASPM, CENPJ, STIL, CEP135, CEP152, ZNF335, PHC1, CDK6, CENPE, SASS6, MFSD2A, ANKLE2, CIT, WDFY3, COPB2, KIF14, NCAPD2, NCAPD3, NCAPH, NUP37*, and *MAP11* (MCPH1–25, respectively) (Table 1). Some of these genes cause microcephaly in combination with other disease features (syndromic microcephaly), however, we will focus on their involvement in primary microcephaly in this review. Because of its heterogeneous nature, diagnosing patients can be a challenging endeavor. Gene panel testing is effective at identifying the more common genetic variants that cause MCPH but becomes impractical with the continual updates required as new genes and variants are discovered. For instance, a recent case report describes a patient with primary microcephaly in which several gene panels were unable to find the disease-causing variant, including the autosomal recessive primary microcephaly Tier 2 sequencing and deletion/duplication panel that screens for several MCPH genes (67). Once whole exome sequencing (WES) was performed on this individual, a homozygous variant in the DNA damage response gene, *TTI2*, was found, representing a novel MCPH locus. Accordingly, it then makes sense to turn to exome or whole genome sequencing (WGS) in order to diagnose patients; however, although these techniques give us a more complete picture of the genome, they do not always immediately provide answers. Exome sequencing of MCPH patients has been shown to have a diagnostic yield of only 29%, with a particular bias for identifying *ASPM* mutations (68). Although that number does increase to about 50% with WGS, this still leaves half of MCPH patients and their families without answers (69). One issue is that WES or WGS typically results in thousands of variants, making the variant prioritization process a daunting endeavor. Therefore, while there is an obvious need to improve our analysis of WES and WGS in order to identify disease-causing variants, it is also necessary to improve our understanding of the causes of MCPH to be able to make new gene–disease associations.

**Table 1:** Overview of OMIM-listed MCPH genes (as of April 2020).

**Gene name**	**Description**	**Cell process**	**Disease ID**	**Inheritance**	**Mode of inactivation**	**Other clinical features**	**Other associated conditions**	**Orthologs**
**CENTRIOLE BIOGENESIS**								
*CENPJ*	Centromere protein J	Centriole biogenesis PCM tethering	MCPH6	AR	Non-sense, frameshift, missense (non-conservative), splicing (3–7)	Facial dysmorphism, developmental delay, joint stiffness, seizures, intellectual disability, cortical malformations, motor problems (3–5)	Seckel syndrome (8)	*sas-4* (*C. elegans*), *Dsas-4* (*Drosophila*), *cenpj* (mouse, zebrafish)
*STIL*	SCL/TAL-interrupting locus	Centriole biogenesis	MCPH7	AR	Non-sense, frameshift, missense (non-conservative), splicing (4, 9–11)	Short stature, seizures, intellectual disability, cortical malformations, visual impairment, motor problems, pre-mature death (3, 4, 11, 12)		*sas-5* (*C. elegans*), *ana2* (*Drosophila*), *stil* (mouse, zebrafish)
*CEP135*	Centrosomal protein 135	Centriole biogenesis	MCPH8	AR	Non-sense, frameshift, splicing (4, 13, 14)	Facial dysmorphism, intellectual disability, cortical malformations, short stature, motor problems, hearing loss (4, 13, 14)		*cep135* (*Drosophila*, mouse, zebrafish), *bld10* (*Chlamydomonas*)
*CEP152*	Centrosomal protein 152	Centriole biogenesis	MCPH9	AR	Missense (non-conservative), non-sense, frameshift, splicing (4, 6, 15, 16)	Cortical malformations, facial dysmorphism, intellectual disability, motor problems (4, 11, 16)	Seckel syndrome (15)	*asterless* (*Drosophila*), *cep152* (mouse, zebrafish)
*SASS6*	Spindle assembly abnormal 6	Centriole biogenesis	MCPH14	AR	Missense (non-conservative) (17)	Intellectual disability, cortical malformations (17)		*sas-6* (*C. elegans, Drosophila*), *bld12p* (*Chlamydomonas*), *sass6* (mouse, zebrafish)
**MICROTUBULE DYNAMICS**								
*WDR62*	WD repeat domain 62	Centriole biogenesis PCM scaffold Microtubule nucleation Spindle orientation	MCPH2	AR	Non-sense, frameshift, missense (non-conservative), splicing (4, 6, 18–23)	Intellectual disability, seizures, motor problems, facial dysmorphism, cortical malformations, developmental delay (3, 18, 19, 22, 23)	Polymicrogyria (18, 22)	*H24G06.1* (*C. elegans*), *wdr62* (*Drosophila*, mouse, zebrafish)
*CDK5RAP2*	CDK5 regulatory subunit-associated protein 2	PCM scaffold Microtubule nucleation Centriolar engagement Cytokinesis Spindle orientation	MCPH3	AR	Non-sense, frameshift, splicing, missense (non-conservative) (7, 24–29)	Hearing loss, leukemia, intellectual disability, short stature, pigmentation abnormalities, facial dysmorphism, cortical malformations (4, 24–31)	Seckel syndrome (24)	*spd-5* (*C. elegans*), *cnn* (*Drosophila*), *cdk5rap2* (mouse, zebrafish)
*KNL1*	Kinetochore scaffold 1	Kinetochore attachment Mitotic checkpoint complex regulator	MCPH4	AR	Splicing, frameshift, missense (non-conservative) (32, 33)	Intellectual disability, cortical malformations, facial dysmorphism, short stature (32, 34, 35)		*knl-1* (*C. elegans*), *knl1* (mouse, zebrafish)
*ASPM*	Abnormal spindle microtubule assembly	Wnt signaling Centriole biogenesis Spindle orientation Cytokinesis	MCPH5	AR	Non-sense, deletion, frameshift, missense (non-conservative), splicing, structural variant (3, 4, 6, 10, 36–40)	Short stature, cortical malformations, heart defects, facial dysmorphism, intellectual disability, pigmentation abnormalities, motor problems, seizures (3, 4, 38–40)		*aspm-1* (*C. elegans*), *asp* (*Drosophila*), *aspm* (mouse, zebrafish)
*CENPE*	Centromere protein E	Kinetochore attachment Mitotic checkpoint complex regulator	MCPH13	AR	Missense (non-conservative) (41)	Facial dysmorphism, seizures, heart defects, intellectual disability, pre-mature death, cortical malformations, motor problems (41)	Microcephalic primordial dwarfism (41)	*cana/cmet* (*Drosophila*), *cenpe* (mouse, zebrafish)
*CIT*	Citron rho-interacting serine/threonine kinase	Microtubule nucleation Cytokinesis Spindle orientation	MCPH17	AR	Missense (non-conservative), splicing, frameshift, non-sense (4, 42–44)	Short stature, intellectual disability, cortical malformations, pre-mature death (42–45)		*W02B8.2* (*C. elegans*), *sticky* (*Drosophila*), *cit* (mouse), *cita/citb* (zebrafish)
*KIF14*	Kinesin 14	Cytokinesis Microtubule network stabilizer	MCPH20	AR	Non-sense, splicing, missense (non-conservative), frameshift (46, 47)	Intellectual disability, speech impairment, developmental delay, motor problems, spasticity, facial dysmorphism, blindness, ADHD, hypotonia (46, 47)	Meckel syndrome (48)	*klp-6* (*C. elegans*), *nebbish* (*Drosophila*), *kif14* (mouse, zebrafish)
*MAP11*	Microtubule-associated protein 11	Cytokinesis Microtubule network stabilizer	MCPH25	AR	Non-sense (49)	Developmental delay, intellectual disability, ADHD, tethered spinal cord (49)		*map11* (mouse, zebrafish)
**DNA DYNAMICS**								
*MCPH1*	Microcephalin	Chromosome condensation Cell cycle checkpoint regulator DNA damage response	MCHP1	AR	Non-sense, deletion, frameshift, missense (non-conservative), splicing (3, 4, 6, 50–53)	Intellectual disability, growth retardation, cortical malformations (50, 54)	Pre-mature chromosome condensation syndrome (50, 54)	*W04A8.1* (*C. elegans*), *mcph1* (*Drosophila*, mouse, zebrafish)
*ZNF335*	Zinc finger protein 335	Transcriptional regulator	MCPH10	AR	Splicing, missense (non-conservative), frameshift (55–57)	Cortical malformations, facial malformations, seizures, hearing loss, motor problems, short stature, pre-mature death (55–57)		*CG8388* (*Drosophila*), *zfp335* (mouse), *znf335* (zebrafish)
*PHC1*	Polyhomeotic homolog 1	Chromatin remodeler	MCPH11	AR	Missense (non-conservative) (58)	Intellectual disability, short stature (58)		*phc1* (mouse, zebrafish)
*ANKLE2*	Ankyrin repeat and lem domain containing 2	Nuclear envelope disassembly	MCPH16	AR	Non-sense, missense (non-conservative) (4, 59)	Cortical malformations, facial dysmorphism, pigmentation abnormalities, motor problems, seizures, vision problems, anemia (4, 59)		*lem-4* (*C. elegans*), *ankle2* (*Drosophila*, mouse, zebrafish)
*NCAPD2*	Non-SMC condensin I complex subunit D2	Chromosome condensation Sister chromatid disentanglement	MCPH21	AR	Splicing, missense (60, 61)	Intellectual disability, growth retardation, short stature (61)		*dpy-28* (*C. elegans*), *cap-d2* (*Drosophila*), *ncapd2* (mouse, zebrafish)
*NCAPD3*	Non-SMC condensin II complex subunit D3	Chromosome condensation Sister chromatid disentanglement	MCPH22	AR	Frameshift, splicing, missense (non-conservative) (61)	Short stature, limb hypertonia, seizures (61)		*hcp-6* (*C. elegans*), *cap-d3* (*Drosophila*), *ncapd3* (mouse, zebrafish)
*NCAPH*	Non-SMC condensin I complex subunit H	Chromosome condensation Sister chromatid disentanglement	MCPH23	AR	Missense (non-conservative) (61)	Intellectual disability (61)		*barren* (*Drosophila*), *ncaph* (mouse, zebrafish)
NUP37	Nucleoporin 37	Nuclear pore complex Kinetochore attachment	MCPH24	AR	Non-sense (61)	Intellectual disability, cortical malformations, clinodactyly (61)		*nup37* (*Drosophila*, mouse, zebrafish)
**SIGNALING**								
*CDK6*	Cyclin-dependent kinase 6	Cell cycle checkpoint regulator	MCPH12	AR	Missense (non-conservative) (62)	Facial dysmorphism, intellectual disability, cortical malformations (62)		*cdk-4* (*C. elegans*), *cdk6* (mouse, zebrafish)
*MFSD2A*	Major facilitator superfamily domain-containing protein 2A	BBB lipid transporter Cell cycle checkpoint regulator	MCPH15	AR	Missense (non-conservative) (4, 63, 64)	Intellectual disability, motor problems, pre-mature death, seizures, cortical malformations (4, 63, 64)		*mfsd2a* (mouse), *mfsd2aa/mfsd2ab* (zebrafish)
*WDFY3*	WD repeat and FYVE domain containing 3	Wnt signaling	MCPH18	AD	Missense (non-conservative) (65)	Intellectual disability (65)		*wdfy-3* (*C. elegans*), *blue cheese* (*Drosophila*), *wdfy3* (mouse, zebrafish)
*COPB2*	Coatamer protein complex subunit beta 2	Cellular trafficking Cell cycle checkpoint regulator	MCPH19	AR	Missense (non-conservative) (66)	Developmental delay, low body weight, blindness, spasticity (66)		*E03H4.8/copb-2* (*C. elegans*), *β'COP* (*Drosophila*), *copb2* (mouse, zebrafish)

*AR, autosomal recessive; AD, autosomal dominant*.

The overall goal of this review is to describe different aspects of MCPH that impact our ability to discover new genes associated with MCPH and, consequently, to diagnose patients. To achieve this goal, we first demonstrate that MCPH arises from perturbations in cell cycle regulation by briefly highlighting the cellular role of microcephaly-associated proteins. Then, we discuss the genetics and evolution of these genes as further considerations in variant prioritization in patients. Throughout this, we provide examples that exemplify how a thorough understanding of the etiology and genetics of disease allows us to identify new disease-causing candidates. Finally, we will comment on a major question in the field—based on what we know about the etiology and genetics of MCPH, why do mutations result in a brain-specific phenotype? Altogether, we illustrate that multidisciplinary approaches facilitate the prioritization of MCPH variants in patients with unknown genetic causes.

## Dissecting the Etiology of Primary Microcephaly

**Figure 1:**
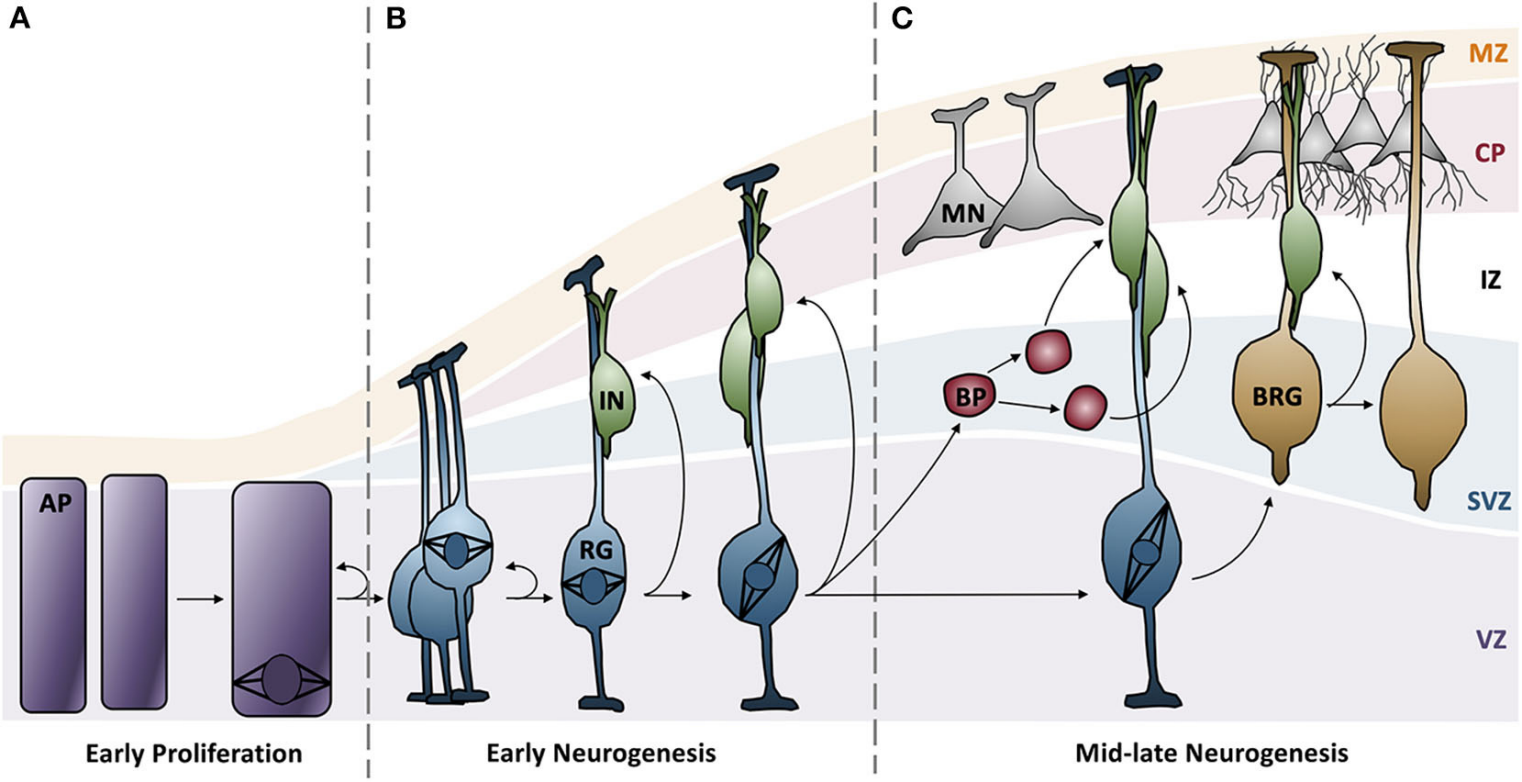
Neurogenesis in the developing neocortex. **(A)** Apical progenitor cells in the ventricular zone undergo symmetrical proliferative divisions, generating a pool of progenitor cells. **(B)** Expression of glial markers causes progenitor cells to differentiate into radial glial cells, which can subsequently undergo symmentrical divisions to generate more radial glial cells or immature neurons. Distinct cortical layers begin to form: ventricular zone, subventricular zone, intermediate zone, cortical plate, and the marginal zone. **(C)** Radial glial cells favor asymmetric division to generate more diverse neuron types and basal progenitors, a secondary progenitor. Radial glial cells continue to differentiate into mature neurons and basal radial glia. AP, apical progenitors; BP, basal progenitors; BRG, basal radial glia; CP, cortical plate; IN, immature neurons; IZ, intermediate zone; MN, mature neurons; MZ, marginal zone; RG, radial glia; SVZ, subventricular zone; VZ, ventricular zone.

MCPH is typically caused by a reduction in the number of neurons in the developing neocortex. Neurons are derived from apical progenitor cells (APs) in the ventricular zone (VZ) of the neocortex (70). In a brief overview, APs divide symmetrically to produce two progenitor cells in the proliferative phase. At the onset of neurogenesis, symmetric cell divisions are favored in order to generate a large pool of progenitor cells; the size of this pool is a good indicator of eventual brain size (Figure 1A) (71). In early neurogenesis phases, APs begin to express glial markers and adopt a radial glial cell (RG) fate; these cells have a highly polarized architecture and are able to divide symmetrically to generate more RGs or young neurons (Figure 1B) (70, 72). As neurogenesis continues, RGs begin to favor asymmetric cell divisions in order to generate more neurons and a secondary progenitor cell, termed basal progenitors (BPs) that localize to the subventricular zone (Figure 1C) (70). BPs serve to amplify the number of neurons that are formed per AP division.

**Figure 2:**
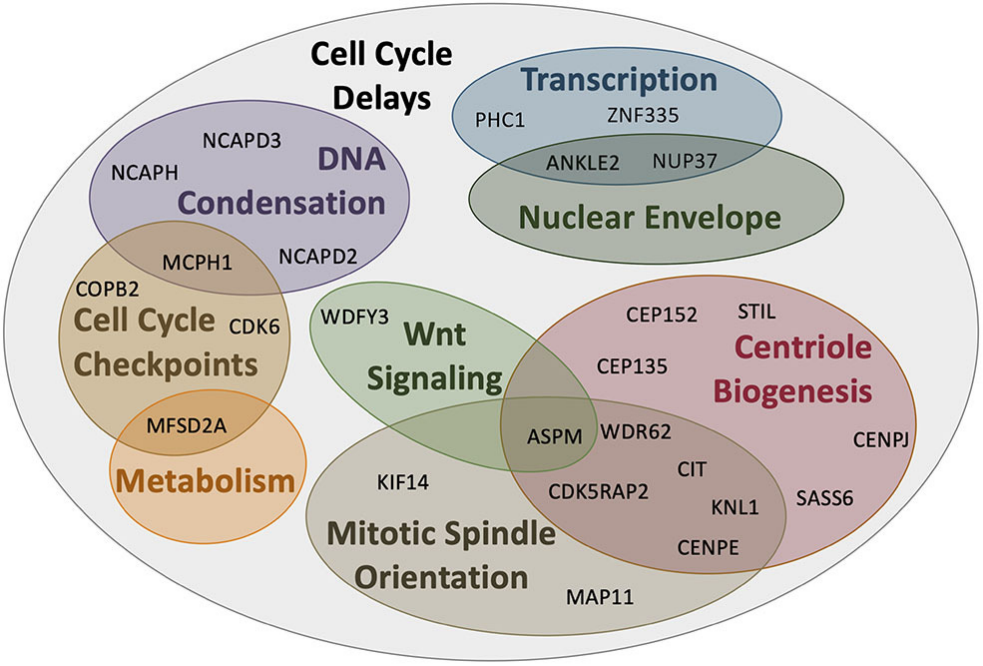
MCPH-associated proteins have overlapping cellular functions that affect cell cycle progression. Aberrant activity in any of these cellular functions would create delays in the timing of the cell cycle and overall proliferation through development. Several proteins act across more than one functional pathway (i.e., centriole biogenesis and mitotic spindle orientation), further delaying the cell cycle at each functionally relevant timepoint.

Based on this developmental model, there are a number of reasons why fewer neurons are produced in MCPH patients. There can be increased cell death of these neurons, an imbalance in the ratio of progenitor to differentiating cells (i.e., changes in asymmetric vs. symmetric cell divisions), changes in the timing of the cell divisions, or abnormal differentiation. However, although each of the MCPH-associated genes affects neuronal population size given their involvement in microcephaly, these genes are involved in a number of different cellular processes that seem rather unconnected. For instance, MCPH genes are involved in centriole biogenesis and regulation, DNA replication and division, cell division, signaling, mitotic spindle orientation, chromosomal condensation, DNA damage responses, microtubule dynamics, and transcriptional control (Figure 2). Despite these seemingly diverse cellular functions, there appears to be a common disease mechanism linking each of these processes—mutations in MCPH genes disrupt the timing of the neurogenic program. This can be modeled using the cell cycle exit fraction, which is the ratio of cells that take on a differentiated fate (i.e., become neurons) vs. those that remain in a proliferative state (73).

To give context to readers that may not be familiar with the cellular function of MCPH genes, this review will begin by demonstrating that mutations in the highlighted genes affect cell cycle timing in some way, effectively increasing the cell cycle exit fraction, indicating that MCPH is a disease arising from cell cycle dysregulation (Figure 2). The cell cycle checkpoints ensure that the cell is appropriately prepared for cell division; they are activated by events such as DNA damage (G2 checkpoint), unattached kinetochores (M checkpoint), limited resources (G1 checkpoint), and/or signaling cues (G1 checkpoint) [reviewed in Pucci et al. (74)]. These checkpoints delay the onset of the next phase to permit the cell to correct any errors that arise. If errors can be repaired, the cell continues to progress through the cell cycle, but if errors are incapable of being fixed, often due to mutations, the cell will often undergo apoptosis to prevent errors from being transmitted (74). Accordingly, not only do ongoing cell cycle delays produce fewer neurons, it is compounded by apoptosis, which further reduces the number of progenitors and neurons within the neocortex. Therefore, a disruption in cell cycle timing appears to be the common mode of pathogenesis underlying MCPH. Although we primarily focus on the described role of each protein encoded by the human MCPH genes to illustrate this model, we also draw from studies of orthologous genes in the murine, nematode, and fly models, as required.

### Centrosomes and the Cell Cycle

**Figure 3:**
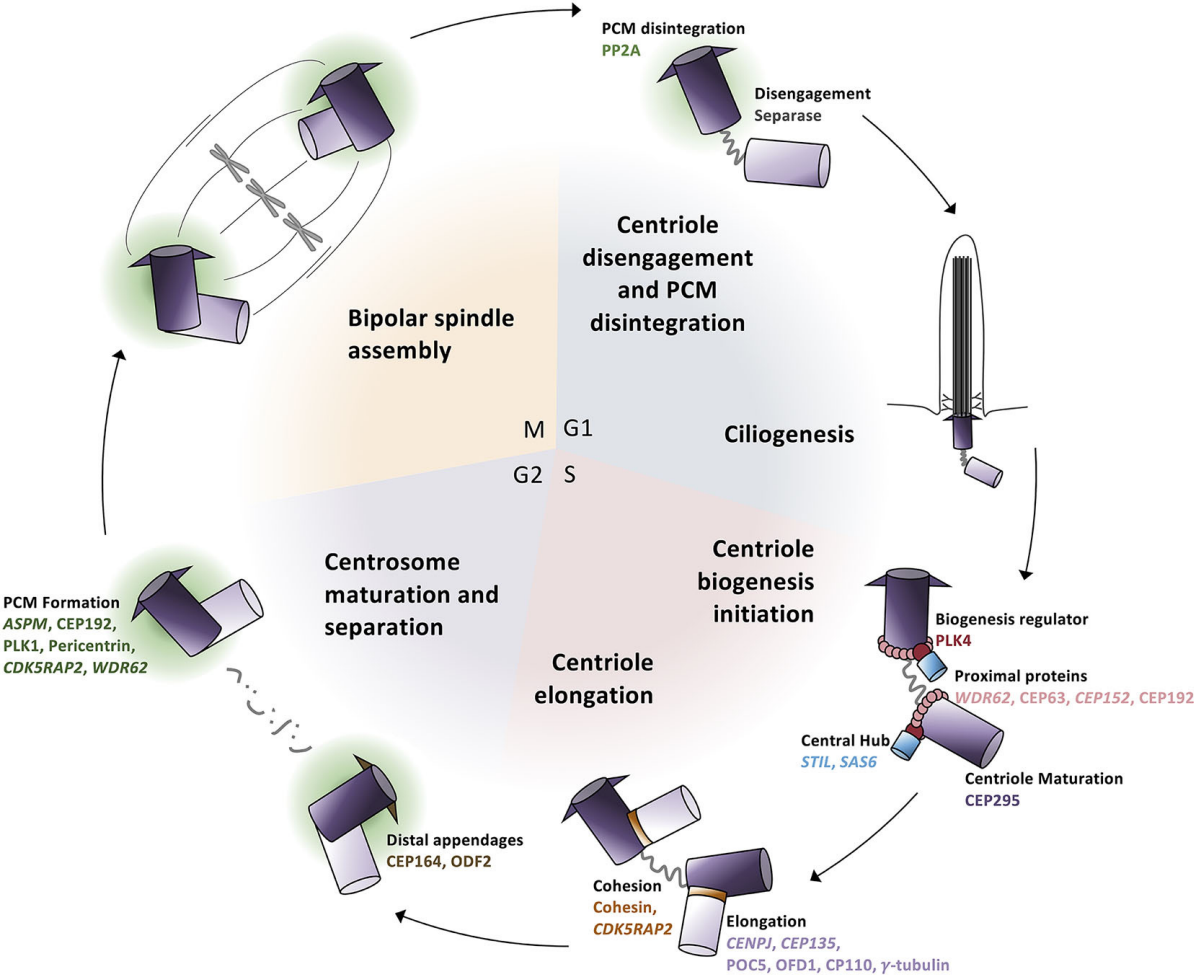
Centrosome biogenesis is linked to the cell cycle. (G1 phase) Centrioles disengage through both separase activity and pericentriolar material (PCM) degradation. The disengaged centriole pair becomes the cilium basal body and acts as the template for ciliogenesis. (S phase) The daughter centriole becomes replication competent and centriole biogenesis is initiated by the recruitment of PLK4 which phosphorylates STIL to begin SAS6 recruitment to generate the central hub. Daughter centrioles elongate and remain attached to the mother centriole via cohesion. (G2 phase) Mother centrioles unlink and centrosome maturation begins with the development of the pericentriolar material and formation of distal appendages. (M phase) Centrosomes travel to opposite poles of the cell for spindle formation and attachment and cell division; each daughter cell contains one centrosome to repeat the cycle.

For many years, MCPH was considered a “centriolopathy” because most of the first genes implicated in causing the disease were involved in centriole biogenesis. Centrosomes are essential for establishing the mitotic spindle during cell division and nucleating the ciliary axoneme during quiescence. Centrosomes are composed of a pair of centrioles (termed mother and daughter centrioles) and associated pericentriolar material (PCM). Centriole biogenesis is tightly linked to the cell cycle (Figure 3). In G1, just after cell division, the two centrioles from one centrosome are loosely linked together. In S phase, this link weakens further and is proceeded by pro-centriole formation (i.e., new daughter centriole biogenesis). In G2, centrosome separation occurs and each new centrosome begins maturation. Finally, in mitosis, each centrosome travels to opposite sides of the cell during spindle formation in preparation for cell division. Therefore, there are four key processes essential in the centrosome cycle that are critical in terms of microcephaly: centriole biogenesis, centriole maturation, centriole tethering, and spindle formation. Variants in genes that are involved in these four key processes have recently been connected with MCPH (Table 1).

#### Centriole Biogenesis

To initiate the formation of daughter centrioles in G1–S phase, the original daughter centriole must first become duplication competent (i.e., must mature into a so-called “mother” centriole). Subsequently, a number of proteins are recruited to the proximal ends of the “grandmother” and “mother” centrioles (Figure 3). The scaffold protein WDR62 recruits CEP63 and CEP152, which form a ring-like structure at the mother centriole's proximal end, and triggers the recruitment of PLK4, a polo-like kinase known as the master regulator of centriole duplication, to the site of daughter centriole formation (75–79).

Following the recruitment of proteins to the mother centriole, PLK4 autophosphorylates itself concurrently with phosphorylating and recruiting STIL (80). PLK4 and STIL subsequently recruit SAS6 to form the template for the nascent daughter centriole (Figure 3). STIL and SAS6 oligomerize into a 9-fold symmetrical ring structure to form the unstable “cartwheel” central hub (80–82). Following central hub formation, proteins such as CENPJ and CEP135 are finally recruited, which aid in regulating and stabilizing central tube elongation and initiating singlet microtubules to assemble around the central hub (80, 83–87).

#### Centriole Maturation

The centrosome-associated pericentriolar material (PCM) is composed of a protein matrix and is responsible for anchoring and nucleating microtubules; just prior to mitosis, this matrix undergoes expansion. Master regulators of PCM maturation recruit coiled-coil proteins as well as the attachment of γ-tubulin to the centrosome by CDK5RAP2; these activities ensure the formation of a PCM scaffold that permits centrosomal microtubule nucleation, which is an essential step in astral microtubule formation (Figure 3) (88–93).

Similar to the centrioles themselves, PCM assembly and disassembly is tightly linked to the cell cycle. During mitotic exit, the dense PCM must disassociate in order to drive centriolar separation (disengagement); daughter centriole biogenesis cannot occur if the so-called “grand-mother” and “mother” centrioles fail to separate because they are entrapped within the PCM (94, 95). Several centrosomal effector molecules therefore become dephosphorylated to promote their destabilization and result in a fragmented PCM, which allows centriole biogenesis to occur (96, 97).

#### Centriole Tethering

Following mitosis, the mother and daughter centriole become disengaged such that the tight cohesin fiber connections between them loosen (95, 98). This requires PCM disintegration and separase protease activity. In G1, disengagement allows the mother centriole to become the basal body in order to nucleate the cilium but also licenses centriole biogenesis in S phase. The newly formed daughter centrioles are tightly bound (engaged) to the mother centriole via cohesin, which is maintained by CDK5RAP2 and functions to limit centriole biogenesis to once per cell cycle (99, 100). Finally, during mitosis, the two engaged centriole pairs (i.e., the centrosomes) lose their loose connection, become separated, and move to opposite sides of the cell where they begin mitotic spindle formation (Figure 3).

#### Spindle Formation

**Figure 4:**
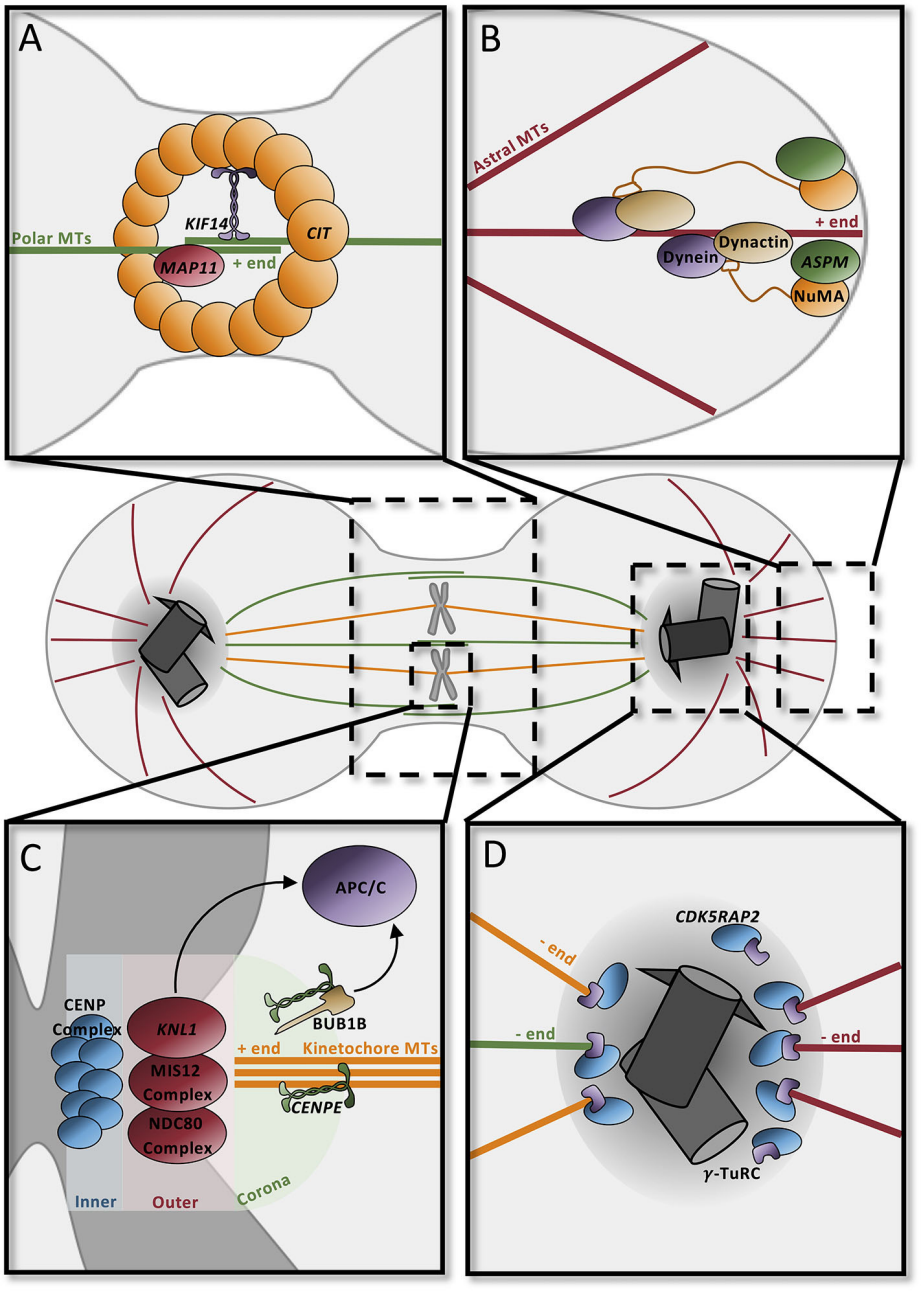
Microtubule dynamics orient the mitotic spindle and drive cell division. **(A)** Contractile ring component CIT at the midbody recruits KIF14 to the central spindle to stabilize the microtubule network. MAP11 promotes cell abscission at the midbody. **(B)** ASPM and NuMA at spindle poles recruit dynein–dynactin to astral microtubules to position spindles in the dividing cell. **(C)** The kinetochore, composed of three distinct layers (inner, outer, and corona), contains several proteins to securely attach microtubules. CENPE in the coronal layer binds microtubules through the motor domain; unbound CENPE signals through BUB1B to the APC/C to delay anaphase. The outer layer complex similarly binds the microtubule positive end; one of the components, KNL1, signals the APC/C to delay cycle if there is improper attachment at this layer. In the inner layer, the CENP complex binds the kinetochore to the condensed chromosome ensuring proper attachment for segregation. **(D)** Pericentriolar material scaffold is formed by CDK5RAP2 for microtubule nucleation by γ-tubulin at the centrosome. APC/C, anaphase promoting complex/cyclosome; MT, microtubules.

The mitotic spindle is responsible for the accurate segregation of chromosomes during cell division. The spindle depends heavily on dynamic microtubule activity for its function: astral microtubules connect the centrosome to the cell cortex, kinetochore microtubules connect the condensed chromosomes to the centrosome, and polar microtubules overlap at the central spindle to connect the two spindle poles (Figure 4). Each microtubule subtype performs specific tasks. Polar microtubules drive the separation of the two centrosomes, direct the positioning of the cleavage furrow, and promote abscission (Figure 4A). Astral microtubules serve to position the mitotic spindle and direct cleavage orientation (Figure 4B), while kinetochore microtubules are responsible for accurately segregating DNA to the opposite poles (Figure 4C). Predictably, many MCPH genes have roles in regulating spindle dynamics, including many of the genes that are involved in centriole biogenesis and PCM maturation (Figure 4D).

During anaphase, the highly abundant polar microtubules emanating from each of the spindle poles overlap centrally in order to form the stable central spindle (Figure 4). This structure is necessary to recruit proteins that position the cleavage furrow and trigger its contraction to form the midbody. Many of the centrosomal proteins that influence microtubule nucleation, such as ASPM, CDK5RAP2, and CIT, are recruited to the midbody during cytokinesis, highlighting the multifunctional roles of these proteins (101). In addition to these proteins, microtubule-associated proteins and their regulators also affect central spindle dynamics. CIT, which is a component of the contractile ring, recruits the microtubule-associated protein KIF14 to the central spindle, where it functions to stabilize the central spindle microtubule network and promote cytokinesis (Figure 4A) (102–105). MAP11 is similarly required at the midbody to promote abscission (49).

The astral microtubules in particular are essential for the orientation of the mitotic spindle (and thus cell division orientation). Rotation of the mitotic spindle depends on forces generated between the cell cortex and the astral microtubules. Minus-end-directed activity of a dynein–dynactin complex, coupled with the cortical anchoring of the astral microtubules, generates a pulling force which allows for rotation and positioning of the spindle (106–108). ASPM seems to be a major player in this process since it binds to nuclear mitotic apparatus (NuMA), which localizes to the spindle poles and the cortex, and subsequently recruits the dynein–dynactin complex to the spindle poles, where it acts as the force generator in spindle positioning (Figure 4B) (109–112).

The kinetochore microtubules are required for the faithful segregation of the chromosomes during cell division; aberrant segregation can lead to chromosomal instabilities or aneuploidy, which can be toxic to the cell. Under the “search and capture model,” microtubules emanating from the spindle poles seek out and attach to the heavily scaffolded kinetochore. The multilayered kinetochore is largely responsible for ensuring this correct attachment and for triggering cellular alarms when microtubules are not appropriately attached (Figure 4D). One of the outermost microtubule-capturing kinetochore components is the centromere-associated protein E (CENPE), which is a large, kinesin-like motor protein that binds to microtubules through its motor domain (113, 114). KNL1, which is located in a complex more interiorly in the kinetochore, is similarly required for microtubule binding (115). If microtubules are incorrectly attached to the kinetochore, then the spindle assembly checkpoint (SAC) will be activated, delaying mitosis until attachment has been corrected. For instance, microtubule-unbound CENPE binds to BUB1B and triggers its phosphorylation which leads to a “delay anaphase” signaling cascade that culminates on the anaphase-promoting complex/cyclosome (APC/C) (114, 116). Similarly, the KNL1 complex functions redundantly with CENPE to delay the cell cycle (117).

### DNA Dynamics During the Cell Cycle

Similar to centrosomes, DNA dynamics are tightly linked to the cell cycle. At the onset of mitosis, one of the cell cycle checkpoints ensures that chromosomes have been accurately condensed and that breakdown of the nuclear envelope occurs; together, these permit the proper pairing of homologous chromosomes at the metaphase plate and the subsequent segregation of sister chromatids into the presumptive daughter cells during anaphase. Following mitosis, cells in G1 become transcriptionally active. Throughout the cell cycle, DNA is continually monitored for damage, which must be repaired in order for the cell to continue to progress through the cycle. Dysregulation of DNA dynamics is thus capable of creating delays in the cell cycle and affecting the overall trajectory of a cell within a developing system, such as the brain. Specifically, delays in the cell cycle of neural progenitors will reduce the proliferative pool and the subsequent number of differentiated neurons within the developing brain. Therefore, because of the tight link between DNA dynamics and cell cycle timing, variants in genes that affect chromosomal condensation, transcriptional regulation, DNA damage responses, and nuclear envelope breakdown are increasingly being implicated in causing MCPH (Table 1).

#### Condensation and Transcriptional Regulation

**Figure 5:**
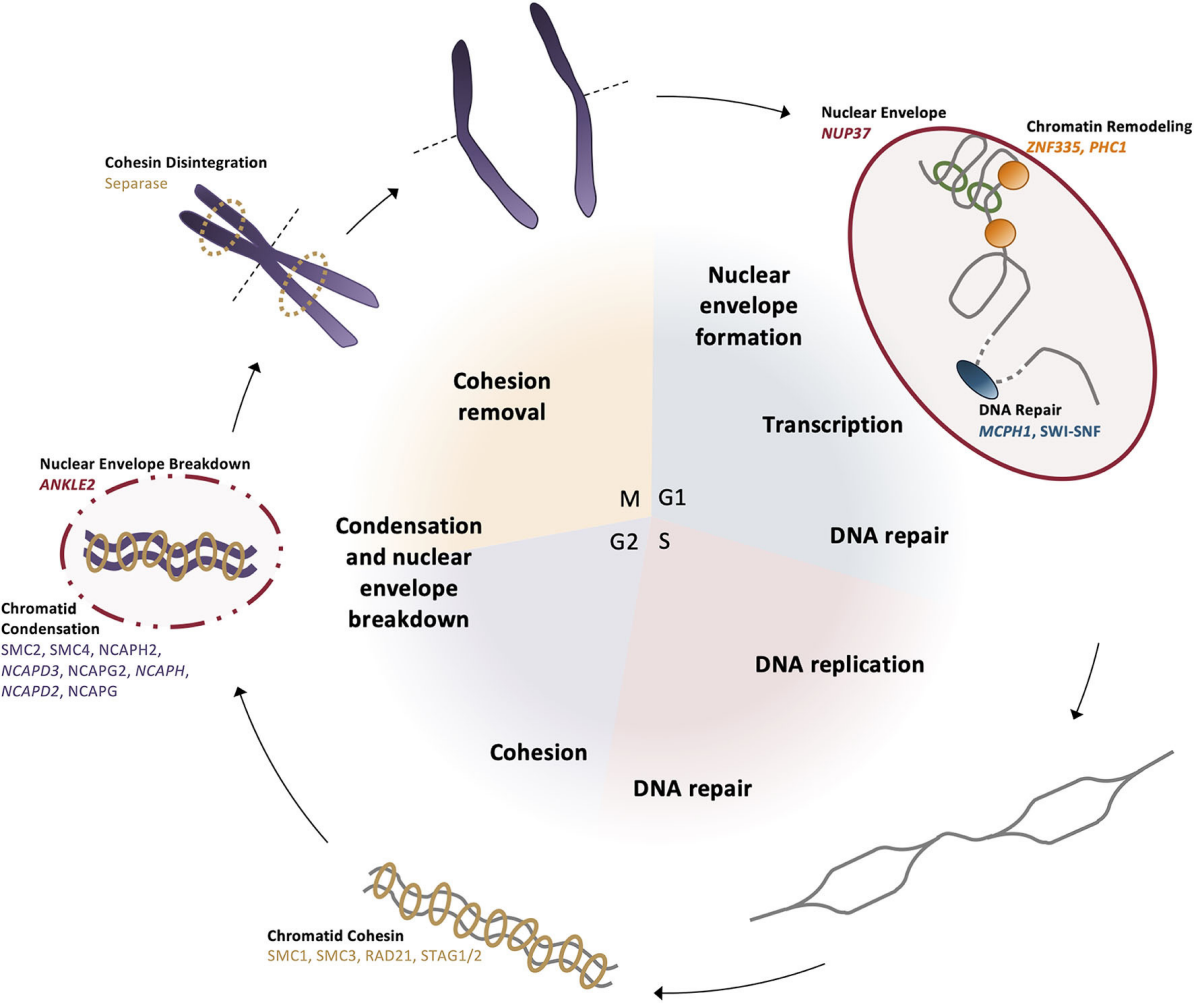
DNA dynamics are linked to the cell cycle. (G1 phase) The nuclear envelope reforms after mitosis, then chromatin is positioned in the nucleus and remodeled for transcription by ZNF335 and PHC1. In preparation for synthesis and the G1 cell cycle checkpoint, DNA repair proteins correct any damage present in the genome. (S phase) Chromosomes undergo replication and repair proteins correct any errors or DNA breaks that occurred during the synthesis process. (G2 phase) Sister chromatids are brought together and bound by cohesin complexes. Prior to mitotic entry, condensin II begins the condensation of the chromatids as the negative regulator MCPH1 is broken down. (M phase) Nuclear envelope is broken down by ANKLE2 and homologous chromosomes align at the metaphase plate. Separase disintegrates the cohesin bonds between sister chromatids so they can be segregated to opposite poles before cytokinesis divides the daughter cells.

Chromosomal condensation is one mode by which transcription is regulated across cell types and throughout the cell cycle. The shift between heterochromatin and euchromatin regulates the genes that are accessible to transcriptional machinery, shifting the transcript profile of each cell. One MCPH gene, *ZNF335*, is an H3K4 methyltransferase that binds to heterochromatin upstream of the neuron-restrictive silencer factor (NRSF) locus in neural stem cells to prevent differentiation pathways; this brain-specific expression pattern is turned off during the dynamic remodeling in the transition to differentiation (Figure 5) (55). PHC1 also represses transcription through chromatin remodeling by ubiquitinating histone H2A to maintain condensation of specific genomic regions (58). Since the switch from proliferation to differentiation requires changes in gene regulation and expression, dynamic chromatin remodeling permits transcriptional machinery binding to various genomic regions related to cell cycle progression and differentiation.

There are many nuclear proteins involved in reducing the length of the chromosomes, some of which are causative in primary microcephaly (118, 119). The primary complexes acting to further condense the chromosomes both laterally and axially are condensin I and condensin II, which share the same structural maintenance core (SMC) subunits but differ in their associated NCAP family subunits (Figure 5). In preparation for entry into mitosis, the SMC associates with NCAPH2, NCAPD3, and NCAPG2 to form condensin II in the nucleus, which localizes to the chromosomes and creates an axially rigid structure of interacting chromosome regions, shortening the overall length of the chromosomes (119, 120). Condensin II localization to the chromosomes is restricted to this phase, as throughout the rest of the cell cycle, it is negatively regulated by MCPH1 until condensation can be coupled with centriole duplication, ensuring that all necessary mitotic structures are formed by the end of G2 (50, 121). Lateral compaction occurs after nuclear envelope breakdown when the condensin I complex—including NCAPH and NCAPD2—can associate with the chromosomes to loop the chromatin around the established axial patterning created by condensin II (Figure 5) (122).

Beginning in S phase, the newly replicated sister chromatids must be connected through to the early mitotic stages, until finally at anaphase, sister chromatids are separated. Sister chromatid cohesion is mediated by the cohesin complexes, which also contain SMC molecules but differ in their accessory protein subunits (Figure 5). This ring-like complex encircles each of the sister chromatid pairs to ensure they are tethered together. At anaphase, the complex is degraded by the protease separase, which is the enzyme that facilitates centriolar disengagement, linking DNA dynamics and centrosome function through the cell cycle (123). This ensures that the sister chromatids are faithfully segregated, and each daughter cell contains identical DNA content. In the event that the cohesin complex pre-maturely dissociates, kinetochore-centromeric attachments fail to occur and genomic instability can result.

#### DNA Damage Response

Prior to synthesis and mitosis, the DNA damage response ensures the accurate passage of genomic information to both daughter cells; if the genome is damaged beyond a reparable threshold, the cell is instead sent into the apoptotic pathway to prevent aberrant cell function or growth and to maintain a healthy cell population. MCPH1 localizes to chromatin that has been damaged by ionizing radiation and prevents compaction of the chromatin at that locus by inhibiting the condensin II complex and, thus, mitotic onset until the repair machinery is able to correct the sequence (Figure 5) (124–126).

#### Nuclear Envelope

In interphase, the nuclear envelope is critical for the movement of proteins into and out of the nucleus, regulating transcription factor access to the genome and the proteins required for the transition to differentiation. NUP37 is a microcephaly-associated nucleoporin that encodes an essential component of the nuclear pore complex (NPC) (Figure 5). It is a component of the ring-shaped Y-complex on the surface of the nuclear envelope, creating much of the structure for the nuclear pore (127). The presence of the Y-complex is necessary for nuclear pore stability and continued proliferation (61). However, during mitosis, the nuclear envelope must disintegrate in order to permit the segregation of the condensed chromosomes. While cells are in interphase, chromatin is bound to the nuclear envelope to maintain each chromosome in its defined region of the nucleus. In preparation for the breakdown of the nuclear envelope during mitosis, ANKLE2 phosphorylates envelope components to reduce the binding affinity between chromatin and the nuclear envelope (128). Once this interaction is broken, the nuclear envelope can begin to disassemble; upon mitotic exit, ANKLE2 dephosphorylates components so the nuclear envelope can reform and reestablish its interaction with the chromatin within the nucleus (128).

### Signaling

The decision for a cell to progress through the cell cycle and divide relies upon numerous signals. For a cell to enter S phase, the cell must assess DNA integrity, metabolic state, and developmental cues—providing these all satisfy certain thresholds, the cell may progress through the cell cycle, which is itself regulated by a series of checkpoints that require the cyclic activity of positive regulators [cyclins and cyclin-dependent kinases (CDKs)] and negative regulators (examples include p53, p21, and retinoblastoma protein). Here, we discuss MCPH genes that influence signaling pathways, such as those involved in cell cycle checkpoints, metabolism, and Wnt signaling.

#### Cell Cycle Regulators

One MCPH gene, *CDK6*, directly regulates the cell cycle; it is responsible for the progression of the cell through G1 phase and the G1/S phase transition (62, 129). In addition, there are two MCPH genes, *COPB2* and *MFSD2A*, that more indirectly affect the cell cycle (66, 130, 131). The first, COPB2, is typically associated with trafficking between the Golgi apparatus and the endoplasmic reticulum, but it has recently been shown to regulate several cell cycle proteins (132). Knockdown of COPB2 increases the expression of CDK inhibitors (P16 and P21) and decreases the expression of cyclin A1 and A2, which are responsible for progression through S phase. *MFSD2A* encodes a fatty acid transporter that acts at the blood–brain barrier (133, 134). It is responsible for the uptake of lysophosphatidylcholines (LPCs), such as those derived from docosahexanoic acid (DHA), which are not synthesized within the brain but are essential for neurogenesis. Specifically, in the early stages of neural stem cell differentiation, DHA promotes cell cycle exit and subsequent differentiation by decreasing the expression of several key cyclins, thus preventing the transition from G1 to S phase (130, 131).

#### Metabolism

MFSDA2 also performs a metabolic role. DHA is a major component of the brain lipid profile as it comprises many of the phospholipids within the brain. Mutations in *MFSD2A* result in increased plasma levels of LPCs and a corresponding decrease in LPC uptake into the brain; this reduced uptake is associated with both lethal and non-lethal microcephaly in humans and animal models (63, 64, 133, 135). The presence of DHA in the brain suppresses the activity of master transcriptional regulators of sterol and fatty acid synthesis (133). Accordingly, reduced uptake of DHA due to mutations in *MFSD2A* results in the increased expression of the master transcriptional regulators and their downstream targets. This indicates that the lipid metabolic pathways must be tightly regulated during neurogenesis, and is highlighted by the link between DHA levels within the brain and proliferation (136).

#### Wnt Signaling

Canonical Wnt signaling activation, at its core, involves the binding of a Wnt-protein ligand to a Frizzled family receptor, which in turn activates the intracellular messenger protein, Disheveled, to influence gene transcription via β-catenin accumulation. Canonical Wnt signaling promotes proliferative cell divisions (i.e., the production of progenitor cells), whereas its loss leads to neuronal differentiation (137–140). ASPM appears to act as a positive regulator of Wnt signaling by preventing the proteasome-mediated degradation of Disheveled (141–143). Conversely, WDFY3 attenuates Wnt signaling; it is responsible for degrading Disheveled aggregates, thus reducing β-catenin levels (65).

## Facilitating Primary Microcephaly Diagnosis

As summarized, MCPH genes are involved in a number of cellular processes, such as centriole biogenesis, mitotic spindle formation, transcription, DNA damage responses, and signaling. Obviously, screening for the 25 known genes in MCPH patients via gene panels or directed sequencing is the simplest method of diagnosing patients and is likely to be successful in about 50% of patients, as previously found (144). However, with advances in WGS, how do we address the remaining 50% of patients? In this section of the review, we discuss a range of aspects to consider when prioritizing variants in patients.

### Linking Cell Biology to Disease

A thorough understanding of the etiology of a disease can guide the diagnostic process. Thus far, we have outlined the molecular roles of each of the MCPH genes known to date, and although each of the thus far implicated genes appear to be involved in distinct cellular processes, MCPH mutations commonly affect the cell cycle exit fraction by pre-maturely increasing the ratio of differentiating to proliferating cells or by triggering apoptosis. For instance, incorrectly positioned mitotic spindles promote asymmetric divisions, thereby increasing the cell cycle exit ratio and cell division delays mimic differentiating cells which have exited the cell cycle. We would therefore predict that mutations in functionally related genes would be likely implicated in the disease. In support of this, a *FZR1* variant, which encodes a component of the APC/C complex and is responsible for driving the cell cycle, has recently been identified in a patient with MCPH-like phenotypes (145). Similarly, a patient with a mutation in *TUBGCP5*, which is a centrosomal component that affects microtubule nucleation and spindle orientation, presents with MCPH (146). Finally, a *METTL5* variant has also recently been identified in a patient with clinical features matching that of MCPH, highlighting the role of epigenetics and transcriptional regulation in the etiology of MCPH (147). Although these three genes are not listed in OMIM as of August 2020 as MCPH genes, we expect that these will be included following database updates and identification of additional patients with mutations in these genes.

### Translation From Genetic Models

One issue when diagnosing patients is that many genes simply do not have a recognized molecular or cellular function ascribed to them. However, the study of these genes in model organisms may reveal a function related to processes disrupted in microcephaly. Not only do most of the MCPH genes have conserved functions across the animal kingdom (Table 1), but mutations in these genes also show a conserved set of phenotypes in model organisms (phenologs); although these phenotypes do not always perfectly mimic the human disease condition, they do reflect perturbation of a common pathway, which gives us a better understanding of whether a given gene is likely to be causal in disease. For instance, modeling MCPH gene mutations in zebrafish, mice, and *Drosophila* typically results in animals with small heads, whereas in *Caenorhabditis elegans*, embryonic lethality with abnormal cell division phenotypes is often observed (49, 65, 69, 80, 83, 148–155). In this way, we can not only validate potential candidates but also begin to screen for possible new candidates that may subsequently be found in human patients. Loss of *cntrob* in zebrafish or *Hmgn2* in mice results in microcephaly, although, as of yet, they are not associated with microcephaly in humans (156, 157). Similarly, pioneering work in *C. elegans* has revealed the importance of SPD-2 (CEP192 in humans) in centriole duplication and PCM maturation, but despite the clear association of other core centriole biogenesis components with microcephaly, CEP192 has not yet been implicated (80, 158). While these genes are not yet associated with MCPH, this may be due to the rarity of these variants in human populations or highly deleterious effects resulting in lethality. Additionally, the phenotypes seen in model organisms may be more profound in the lab environment than they would present in humans, decreasing their chances of being discovered.

### Analyzing Inheritance Patterns

Another mechanism of prioritizing variants is to examine the inheritance pattern. With the exception of *WDFY3*, all MCPH variants are autosomal recessively inherited (Table 1). Many microcephaly patients exhibit homozygous variants in the disease-causing gene due to consanguinity, but increasingly, compound heterozygous variants are being discovered. Therefore, in non-consanguineous families, performing triad sequencing and using filtering methods that include compound heterozygous variants are essential. However, recent evidence suggests that the inheritance of MCPH may be more complicated than originally thought and alternative forms of inheritance should be considered in difficult-to-diagnose patients. For instance, similar to *WDFY3*, mutations in *DPP6*, which encodes a dipeptidyl peptidase protein, cause autosomal dominant microcephaly in addition to intellectual disabilities, indicating that it may also be necessary to screen through heterozygous variants in patients (159). Furthermore, there is increasing evidence that MCPH may occasionally follow an oligogenic inheritance pattern; a recent study found that microcephaly patients have a higher mutational burden in genes implicated in causing MCPH (69). Additionally, the digenic inheritance of *CDK5RAP2* and *CEP152* heterozygous mutations causes Seckel syndrome, a disease which includes microcephaly as a clinical feature (24). Again, turning to genetic models may validate instances where digenic inheritance is predicted to be necessary for phenotypic presentation or where modifying genes affect phenotypic severity. In the fish model, for example, homozygous mutations in both *aspm* and *wdr62* are necessary to produce the microcephaly phenotype (69). Considering that ciliopathies are well-established digenic conditions, and like microcephalies, depend upon proper cell cycle timing and centrosome function (Figure 3), it is not challenging to envision a model in which these diseases genetically parallel each other. Conversely, a mutation in *Ttc21b* causes genetic background-dependent microcephaly in mouse; quantitative trait locus analysis revealed a missense mutation in one genetic background but not the other that was able to enhance the *Ttc21b* neural phenotypes (160). Therefore, clinicians may have to look past traditional autosomal recessively inherited variants in a single gene in particularly challenging to diagnose patients.

### Determining Mode of Gene Inactivation

Intriguingly, most of the MCPH-causing mutations are non-sense, frameshift, or splicing mutations, all of which typically result in a complete loss-of-function of the protein product (Table 1). However, this also implies that other types of nullifying mutations, such as structural variants, copy number variants (CNVs), or non-coding regulatory variants, may be causative in microcephaly. Already, larger deletion mutations in *MCPH1* and *ASPM* have been identified in MCPH patients, in addition to a translocation break in *ASPM* that disrupts the coding sequence (3, 36, 37, 51). However, these types of variations are likely to be increasingly discovered as our methods of identifying these variants improve. Similarly, the use of transcriptomics, such as RNA-seq, may expedite our identification of non-coding variants in patients; the use of transcriptomics in a patient with microcephaly-micromelia syndrome found a non-coding mutation in *DONSON* that caused aberrant splicing (161). Multimethod approaches to identifying causal mutations may therefore be necessary, in addition to using tools that are capable of detecting a wide range of variants.

### Evolution of Brain Size in Primates

One of the hallmarks of human evolution is an increase in brain size and complexity, a change that was accompanied by increased cognitive power. Interestingly, there is a correlation between genes predicted to be responsible for driving the increase in human brain size and those that are implicated in causing MCPH. For instance, *MCPH1, CDK5RAP2, CENPJ*, and *ASPM* have been shown to have undergone positive selection during primate evolution (162–165). Researchers speculate that over the course of evolution, MCPH genes may have accumulated genetic changes that permitted the increase in hominid brain size—specifically, these changes likely altered the rate of cell division in proliferating neuronal stem cells. Conversely, the disease state may be an example of atavism, in which nullifying mutations recreate a more ancestral state. Another possibility is the changes in copy number may underlie hominid brain size changes; a recently discovered example is *NOTCH2NL*, in which three copies are necessary for normal human development (166). The duplication or deletion of the locus in which *NOTCH2NL* is located results in macrocephaly or microcephaly, respectively. Therefore, evolutionary analyses, such as those determining whether a gene undergoes positive selection through the course of hominid brain evolution, may be one more mechanism by which microcephaly-causing variants are prioritized [reviewed in Gilbert et al. (166)].

## Remaining Questions

*Why do mutations in MCPH genes specifically affect neurogenesis?* While some MCPH genes have enriched expression in neural progenitors or have a biological function limited to the brain (e.g., *MFSD2A*), many genes are ubiquitously expressed and have biological functions that are necessary in many tissue types (18, 134). Therefore, a major question is why mutations in the MCPH genes, which are typically nullifying mutations, specifically cause microcephaly, with generally few abnormalities outside of the head and brain. This is especially true when we consider that in the *C. elegans* model, mutations in orthologous MCPH genes result in lethality, which would be predicted based on the essential function of these genes in cell division. One similarity between neuroprogenitors and *C. elegans* development though is that the cell cycle length is remarkably short; in humans, the length is shorter in comparison to other cell types, and in *C. elegans*, the first few cell divisions occur extremely rapidly and are stereotypically oriented (167, 168). In fact, G1 length has been shown to be an essential regulator of the switch between proliferation and differentiation, which explains why mutations in MCPH genes, which generally cause a lengthening in the cell cycle and thus pre-maturely increase the cell cycle exit fraction, have dramatic effects on the number of progenitors remaining in a proliferative state (129, 169). The brain therefore appears to be more sensitive to changes in cell cycle length relative to other tissue types, an effect that may be appropriately modeled using the *C. elegans* genetic system.

Another possibility may lie in alternative splicing. The brain undergoes the most alternative splicing events than any other tissue and expresses the largest number of splicing factor genes (170). This means that the brain produces more diverse protein isoforms than other tissue types. Accordingly, there are many examples of splicing mutations in MCPH genes (Table 1); it is possible that the disruption in splicing in these genes affects the protein isoforms that are uniquely expressed in the brain. For instance, a splicing mutation in *KNL1* specifically affects neuronal progenitor cells, but fibroblasts and neural crest cells expressing the same mutation are unaffected (171). Although not every MCPH mutation affects splicing, this may be one mechanism that contributes to the brain-specific presentation.

Finally, the classical clinical definition of MCPH describes an isolated disorder affecting the head and brain size, with typically no other malformations. However, as more patients continue to be discovered and diagnosed with MCPH, the clinical landscape of primary microcephaly expands. We now know that in addition to a small head, MCPH is frequently associated with cortical and facial malformations, intellectual disabilities, and seizures, in addition to short stature, heart problems, and in extreme cases, pre-mature death (Table 1). Many of the genes are also implicated in causing different but related diseases, such as Seckel syndrome or microcephalic primordial dwarfism, indicating that there is likely an overlapping pathophysiology between these conditions, and they may represent a disease spectrum rather than individual conditions. In support of this, many genes that are implicated in causing syndromic microcephaly affect the same pathways as MCPH genes, indicating that cell cycle dysregulation underlies both isolated and syndromic microcephalies. This reflects the prediction that mutations in genes affecting cell cycle progression would present with more pleiotropic effects.

## Concluding Remarks

MCPH is a heterogeneous disorder that, like many rare diseases, has been challenging to diagnose despite advances in genomics. Therefore, there is a need to understand the genetics and cell biology underlying the disease in order to expedite discovery and genetic diagnosis. This review has shown that microcephaly is caused by aberrant cell cycle regulation by summarizing the molecular functions of each of the known MCPH-causing genes and their associated cellular phenotypes, as well as providing examples of novel MCPH candidates. In addition to furthering our understanding of the disease pathogenesis, we have also provided insights on the genetics and inheritance of MCPH with the hopes of facilitating the variant prioritization process in patients with microcephaly. Altogether, the incorporation of each of these disease facets in the process of identifying the MCPH-causing gene(s) will improve diagnosis rates in these patients and will guide family planning and personalized treatments but also forms a template for the inclusion of multidisciplinary approaches in the diagnostic process.

## Author Contributions

FJ and MT-G: conceptualization and funding acquisition. FJ and AS: writing—original draft preparation. MT-G: writing—review and editing and supervision. All authors have read and agreed to the final manuscript version.

## Conflict of Interest

The authors declare that the research was conducted in the absence of any commercial or financial relationships that could be construed as a potential conflict of interest.

## References

[1] Cox J Jackson AP Bond J Woods CG. What primary microcephaly can tell us about brain growth. Trends Mol Med. (2006) 12:358–66. 10.1016/j.molmed.2006.06.006 16829198

[2] Van Den Bosch J. Microcephaly in the Netherlands: a clinical and genetical study. Ann Hum Genet. (1959) 23:91–116. 10.1111/j.1469-1809.1958.tb01455.x 13637554

[3] Garshasbi M Motazacker MM Kahrizi K Behjati F Abedini SS Nieh SE. SNP array-based homozygosity mapping reveals MCPH1 deletion in family with autosomal recessive mental retardation and mild microcephaly. Hum Genet. (2006) 118:708–15. 10.1007/s00439-005-0104-y 16311745

[4] Gul A Hassan MJ Hussain S Raza SI Chishti MS Ahmad W. A novel deletion mutation in CENPJ gene in a Pakistani family with autosomal recessive primary microcephaly. J Hum Genet. (2006) 51:760–4. 10.1007/s10038-006-0017-1 16900296

[5] Sajid Hussain M Marriam Bakhtiar S Farooq M Anjum I Janzen E Reza Toliat M. Genetic heterogeneity in Pakistani microcephaly families. Clin Genet. (2013) 83:446–51. 10.1111/j.1399-0004.2012.01932.x 22775483

[6] Bond J Roberts E Springell K Lizarraga S Scott S Higgins J. A centrosomal mechanism involving CDK5RAP2 and CENPJ controls brain size. Nat Genet. (2005) 37:353–5. 10.1038/ng1539 15793586

[7] Al-Dosari MS Shaheen R Colak D Alkuraya FS. Novel CENPJ mutation causes Seckel syndrome. J Med Genet. (2010) 47:411–4. 10.1136/jmg.2009.076646 20522431

[8] Kumar A Girimaji SC Duvvari MR Blanton SH. Mutations in STIL, encoding a pericentriolar and centrosomal protein, cause primary microcephaly. Am J Hum Genet. (2008) 84:286–90. 10.1016/j.ajhg.2009.01.017 19215732 PMC2668020

[9] Papari E Bastami M Farhadi A Abedini S Hosseini M Bahman I. Investigation of primary microcephaly in Bushehr province of Iran: novel *STIL* and *ASPM* mutations. Clin Genet. (2013) 83:488–90. 10.1111/j.1399-0004.2012.01949.x 22989186

[10] Kakar N Ahmad J Morris-Rosendahl DJ Altmüller J Friedrich K Barbi G. STIL mutation causes autosomal recessive microcephalic lobar holoprosencephaly. Hum Genet. (2014) 134:45–51. 10.1007/s00439-014-1487-4 25218063

[11] Cristofoli F De Keersmaecker B De Catte L Vermeesch JR Van Esch H. Novel STIL compound heterozygous mutations cause severe fetal microcephaly and centriolar lengthening. Mol Syndromol. (2017) 8:282–93. 10.1159/000479666 29230157 PMC5701267

[12] Farooq M Fatima A Mang Y Hansen L Kjaer KW Baig SM. A novel splice site mutation in CEP135 is associated with primary microcephaly in a Pakistani family. J Hum Genet. (2016) 61:271–3. 10.1038/jhg.2015.138 26657937

[13] Hussain MS Baig SM Neumann S Nürnberg G Farooq M Ahmad I. A truncating mutation of CEP135 causes primary microcephaly and disturbed centrosomal function. Am J Hum Genet. (2012) 90:871–8. 10.1016/j.ajhg.2012.03.016 22521416 PMC3376485

[14] Kalay E Yigit G Aslan Y Brown KE Pohl E Bicknell LS. CEP152 is a genome maintenance protein disrupted in Seckel syndrome. Nat Genet. (2011) 43:23–6. 10.1038/ng.725 21131973 PMC3430850

[15] Guernsey DL Jiang H Hussin J Arnold M Bouyakdan K Perry S. Mutations in centrosomal protein CEP152 in primary microcephaly families linked to MCPH4. Am J Hum Genet. (2010) 87:40–51. 10.1016/j.ajhg.2010.06.003 20598275 PMC2896783

[16] Khan MA Rupp VM Orpinell M Hussain MS Altmüller J Steinmetz MO. A missense mutation in the PISA domain of HsSAS-6 causes autosomal recessive primary microcephaly in a large consanguineous Pakistani family. Hum Mol Genet. (2014) 23:5940–9. 10.1093/hmg/ddu318 24951542

[17] Yi YG Lee D-W Kim J Jang J-H Lee S-M Jang D-H. Two novel mutations (c.883-4_890del and c.1684C&gt;G) of WDR62 gene associated with autosomal recessive primary microcephaly: a case report. Front Pediatr. (2019) 7:457. 10.3389/fped.2019.00457 31788460 PMC6854001

[18] Rakic P. A small step for the cell, a giant leap for mankind: a hypothesis of neocortical expansion during evolution. Trends Neurosci. (1995) 18:383–8. 10.1016/0166-2236(95)93934-P 7482803

[19] Nicholas AK Khurshid M Désir J Carvalho OP Cox JJ Thornton G. WDR62 is associated with the spindle pole and is mutated in human microcephaly. Nat Genet. (2010) 42:1010–4. 10.1038/ng.682 20890279 PMC5605390

[20] Yu TW Mochida GH Tischfield DJ Sgaier SK Flores-Sarnat L Sergi CM. Mutations in WDR62, encoding a centrosome-associated protein, cause microcephaly with simplified gyri and abnormal cortical architecture Human Splicing Finder Version 2.3 HHS Public Access Author manuscript. Nat Genet. (2010) 42:1015–20. 10.1038/ng.683 20890278 PMC2969850

[21] Murdock DR Clark GD Bainbridge MN Newsham I Wu Y-Q Muzny DM Whole-exome sequencing identifies compound heterozygous mutations in WDR62 in siblings with recurrent polymicrogyria. Am J Med Genet Part A. (2011) 155:2071–7. 10.1002/ajmg.a.34165 PMC3616765 21834044

[22] Bhat V Girimaji S Mohan G Arvinda H Singhmar P Duvvari M. Mutations in WDR62, encoding a centrosomal and nuclear protein, in Indian primary microcephaly families with cortical malformations. Clin Genet. (2011) 80:532–40. 10.1111/j.1399-0004.2011.01686.x 21496009

[23] Jouan L Bencheikh BOA Daoud H Dionne-Laporte A Dobrzeniecka S Spiegelman D. Exome sequencing identifies recessive CDK5RAP2 variants in patients with isolated agenesis of corpus callosum. Eur J Hum Genet. (2016) 24:607–10. 10.1038/ejhg.2015.156 26197979 PMC4929875

[24] Snedeker J Gibbons WJ Paulding DF Abdelhamed Z Prows DR Stottmann RW. Gpr63 is a modifier of microcephaly in Ttc21b mouse mutants. PLoS Genet. (2019) 15:e1008467. 10.1371/journal.pgen.1008467 31730647 PMC6881074

[25] Pagnamenta AT Murray JE Yoon G Akha ES Harrison V Bicknell LS A novel nonsense CDK5RAP2 mutation in a Somali child with primary microcephaly and sensorineural hearing loss. Am J Med Genet Part A. (2012) 158:2577–82. 10.1002/ajmg.a.35558 PMC3470702 22887808

[26] Lancaster MA Renner M Martin CA Wenzel D Bicknell LS Hurles ME. Cerebral organoids model human brain development and microcephaly. Nature. (2013) 501:373–9. 10.1038/nature12517 23995685 PMC3817409

[27] Tan CA Topper S Ward Melver C Stein J Reeder A Arndt K. The first case of CDK5RAP2-related primary microcephaly in a non-consanguineous patient identified by next generation sequencing. Brain Dev. (2014) 36:351–5. 10.1016/j.braindev.2013.05.001 23726037

[28] Pagnamenta AT Howard MF Knight SJL Keays DA Quaghebeur G Taylor JC. Activation of an exonic splice-donor site in exon 30 of *CDK5RAP2* in a patient with severe microcephaly and pigmentary abnormalities. Clin Case Reports. (2016) 4:952–6. 10.1002/ccr3.663 27761245 PMC5054469

[29] Moynihan L Jackson AP Roberts E Karbani G Lewis I Corry P. A third novel locus for primary autosomal recessive microcephaly maps to chromosome 9q34. Am J Hum Genet. (2000) 66:724–7. 10.1086/302777 10677332 PMC1288125

[30] Heney D Mueller R Turner G Karbani G Cadranel J Lewis IJ. Familial microcephaly with normal intelligence in a patient with acute lymphoblastic leukemia. Cancer. (1992) 69:962–5. 10.1002/1097-0142(19920215)69:4<962::AID-CNCR2820690421>3.0.CO;2-W 1735087

[31] Zarate YA Kaylor JA Bosanko K Lau S Vargas J Gao H. First clinical report of an infant with microcephaly and *CASC5* mutations. Am J Med Genet Part A. (2016) 170:2215–8. 10.1002/ajmg.a.37726 27149178

[32] Genin A Desir J Lambert N Biervliet M Van Der Aa N Pierquin G. Kinetochore KMN network gene CASC5 mutated in primary microcephaly. Hum Mol Genet. (2012) 21:5306–17. 10.1093/hmg/dds386 22983954

[33] Saadi A Verny F Siquier-Pernet K Bole-Feysot C Nitschke P Munnich A. Refining the phenotype associated with CASC5 mutation. Neurogenetics. (2016) 17:71–8. 10.1007/s10048-015-0468-7 26626498

[34] Jamieson CR Govaerts C Abramowicz MJ. Primary autosomal recessive microcephaly: homozygosity mapping of MCPH4 to chromosome 15. Am J Hum Genet. (1999) 65:1465–9. 10.1086/302640 10521316 PMC1288302

[35] Létard P Drunat S Vial Y Duerinckx S Ernault A Amram D. Autosomal recessive primary microcephaly due to *ASPM* mutations: an update. Hum Mutat. (2018) 39:319–32. 10.1002/humu.23381 29243349

[36] Pichon B Vankerckhove S Bourrouillou G Duprez L Abramowicz MJ. A translocation breakpoint disrupts the ASPM gene in a patient with primary microcephaly. Eur J Hum Genet. (2004) 12:419–21. 10.1038/sj.ejhg.5201169 14997185

[37] Evrony GD Cordero DR Shen J Partlow JN Yu TW Rodin RE. Integrated genome and transcriptome sequencing identifies a noncoding mutation in the genome replication factor DONSON as the cause of microcephaly-micromelia syndrome. Genome Res. (2017) 27:1323–35. 10.1101/gr.219899.116 28630177 PMC5538549

[38] Bond J Scott S Hampshire DJ Springell K Corry P Abramowicz MJ. Protein-truncating mutations in ASPM cause variable reduction in brain size. Am J Hum Genet. (2003) 73:1170–7. 10.1086/379085 14574646 PMC1180496

[39] Shen J Eyaid W Mochida GH Al-Moayyad F Bodell A Woods CG. ASPM mutations identified in patients with primary microcephaly and seizures. J Med Genet. (2005) 42:725–9. 10.1136/jmg.2004.027706 16141009 PMC1736131

[40] Mirzaa GM Vitre B Carpenter G Abramowicz I Gleeson JG Paciorkowski AR. Mutations in CENPE define a novel kinetochore-centromeric mechanism for microcephalic primordial dwarfism. Hum Genet. (2014) 133:1023–39. 10.1007/s00439-014-1443-3 24748105 PMC4415612

[41] Harding BN Moccia A Drunat S Soukarieh O Ne Tubeuf H Chitty LS. Mutations in citron kinase cause recessive microlissencephaly with multinucleated neurons. Am J Hum Genet. (2016) 99:511–20. 10.1016/j.ajhg.2016.07.003 27453579 PMC4974106

[42] Basit S Al-Harbi KM Alhijji SAM Albalawi AM Alharby E Eldardear A. CIT, a gene involved in neurogenic cytokinesis, is mutated in human primary microcephaly. Hum Genet. (2016) 135:1199–207. 10.1007/s00439-016-1724-0 27519304

[43] Li H Bielas SL Zaki MS Ismail S Farfara D Um K. Biallelic mutations in citron kinase link mitotic cytokinesis to human primary microcephaly. Am J Hum Genet. (2016) 99:501–10. 10.1016/j.ajhg.2016.07.004 27453578 PMC4974110

[44] Shaheen R Hashem A Abdel-Salam GMH Al-Fadhli F Ewida N Fowzan SA. Mutations in CIT, encoding citron rho-interacting serine/ threonine kinase, cause severe primary microcephaly in humans. Hum Genet. (2016) 135:1191–7. 10.1007/s00439-016-1722-2 27503289

[45] Moawia A Shaheen R Rasool S Waseem SS Ewida N Budde B. Mutations of KIF14 cause primary microcephaly by impairing cytokinesis. Ann Neurol. (2017) 82:562–77. 10.1002/ana.25044 28892560

[46] Makrythanasis P Maroofian R Stray-Pedersen A Musaev D Zki MS Mahmoud IG. Biallelic variants in KIF14 cause intellectual disability with microcephaly. Eur J Hum Genet. (2018) 26:330–9. 10.1038/s41431-017-0088-9 29343805 PMC5839044

[47] Filges I Nosova E Bruder E Tercanli S Townsend K Gibson WT. Exome sequencing identifies mutations in KIF14 as a novel cause of an autosomal recessive lethal fetal ciliopathy phenotype. Clin Genet. (2014) 86:220–8. 10.1111/cge.12301 24128419

[48] Farooq M Baig S Tommerup N Kjaer KW Craniosynostosis-microcephaly with chromosomal breakage and other abnormalities is caused by a truncating MCPH1 mutation and is allelic to premature chromosomal condensation syndrome and primary autosomal recessive microcephaly type 1. Am J Med Genet Part A. (2010) 152:495–7. 10.1002/ajmg.a.33234 20101680

[49] Perez Y Bar-Yaacov R Kadir R Wormser O Shelef I Birk OS. Mutations in the microtubule-associated protein MAP11 (C7orf43) cause microcephaly in humans and zebrafish. Brain. (2019) 142:574–85. 10.1093/brain/awz004 30715179 PMC6391606

[50] Trimborn M Bell SM Felix C Rashid Y Jafri H Griffiths PD. Mutations in microcephalin cause aberrant regulation of chromosome condensation. Am J Hum Genet. (2004) 75:261–6. 10.1086/422855 15199523 PMC1216060

[51] Nicholas AK Swanson EA Cox JJ Karbani G Malik S Springell K. The molecular landscape of ASPM mutations in primary microcephaly. J Med Genet. (2009) 46:249–53. 10.1136/jmg.2008.062380 19028728 PMC2658750

[52] Jackson AP Eastwood H Bell SM Adu J Toomes C Carr IM. Identification of microcephalin, a protein implicated in determining the size of the human brain. Am J Hum Genet. (2002) 71:136–42. 10.1086/341283 12046007 PMC419993

[53] Neitzel H Neumann LM Schindler D Wirges A Tönnies H Trimborn M. Premature chromosome condensation in humans associated with microcephaly and mental retardation: a novel autosomal recessive condition. Am J Hum Genet. (2002) 70:1015–22. 10.1086/339518 11857108 PMC379095

[54] Sato R Takanashi JI Tsuyusaki Y Kato M Saitsu H Matsumoto N. Association between invisible Basal Ganglia and ZNF335 mutations: a case report. Pediatrics. (2016) 138:e20160897. 10.1542/peds.2016-0897 27540107

[55] Yang YJ Baltus AE Mathew RS Murphy EA Evrony GD Gonzalez DM. Microcephaly gene links trithorax and REST/NRSF to control neural stem cell proliferation and differentiation. Cell. (2012) 151:1097–112. 10.1016/j.cell.2012.10.043 23178126 PMC3567437

[56] Stouffs K Stergachis AB Vanderhasselt T Dica A Janssens S Vandervore L. Expanding the clinical spectrum of biallelic ZNF335 variants. Clin Genet. (2018) 94:246–51. 10.1111/cge.13260 29652087 PMC6361164

[57] Yamamoto S Jaiswal M Charng WL Gambin T Karaca E Mirzaa G. A drosophila genetic resource of mutants to study mechanisms underlying human genetic diseases. Cell. (2014) 159:200–14. 10.1016/j.cell.2014.09.002 25259927 PMC4298142

[58] Awad S Al-Dosari MS Al-Yacoub N Colak D Salih MA Alkuraya FS. Mutation in PHC1 implicates chromatin remodeling in primary microcephaly pathogenesis. Hum Mol Genet. (2013) 22:2200–13. 10.1093/hmg/ddt072 23418308

[59] Reuter MS Tawamie H Buchert R Gebril OH Froukh T Thiel C. Diagnostic yield and novel candidate genes by exome sequencing in 152 consanguineous families with neurodevelopmental disorders. JAMA Psychiatry. (2017) 74:293–9. 10.1001/jamapsychiatry.2016.3798 28097321

[60] Martin CA Murray JE Carroll P Leitch A Mackenzie KJ Halachev M. Mutations in genes encoding condensin complex proteins cause microcephaly through decatenation failure at mitosis. Genes Dev. (2016) 30:2158–72. 10.1101/gad.286351.116 27737959 PMC5088565

[61] Braun DA Lovric S Schapiro D Schneider R Marquez J Asif M. Mutations in multiple components of the nuclear pore complex cause nephrotic syndrome. J Clin Invest. (2018) 128:4313–28. 10.1172/JCI98688 30179222 PMC6159964

[62] Hussain MS Baig SM Neumann S Peche VS Szczepanski S Nürnberg G. CDK6 associates with the centrosome during mitosis and is mutated in a large pakistani family with primary microcephaly. Hum Mol Genet. (2013) 22:5199–214. 10.1093/hmg/ddt374 23918663

[63] Alakbarzade V Hameed A Quek DQY Chioza BA Baple EL Cazenave-Gassiot A. A partially inactivating mutation in the sodium-dependent lysophosphatidylcholine transporter MFSD2A causes a non-lethal microcephaly syndrome. Nat Genet. (2015) 47:814–7. 10.1038/ng.3313 26005865

[64] Guemez-Gamboa A Nguyen LN Yang H Zaki MS Kara M Ben-Omran T. Inactivating mutations in MFSD2A, required for omega-3 fatty acid transport in brain, cause a lethal microcephaly syndrome. Nat Genet. (2015) 47:809–13. 10.1038/ng.3311 26005868 PMC4547531

[65] Kadir R Harel T Markus B Perez Y Bakhrat A Cohen I. ALFY-Controlled DVL3 autophagy regulates wnt signaling, determining human brain size. PLoS Genet. (2016) 12:e1005919. 10.1371/journal.pgen.1005919 27008544 PMC4805177

[66] DiStasio A Driver A Sund K Donlin M Muraleedharan RM Pooya S. Copb2 is essential for embryogenesis and hypomorphic mutations cause human microcephaly. Hum Mol Genet. (2017) 26:4836–48. 10.1093/hmg/ddx362 29036432 PMC5886270

[67] Picher-Martel V Labrie Y Rivest S Lace B Chrestian N. Whole-exome sequencing identifies homozygous mutation in TTI2 in a child with primary microcephaly: a case report. BMC Neurol. (2020) 20:58. 10.1186/s12883-020-01643-1 32061250 PMC7023720

[68] Rump P Jazayeri O van Dijk-Bos KK Johansson LF van Essen AJ Verheij JBGM. Whole-exome sequencing is a powerful approach for establishing the etiological diagnosis in patients with intellectual disability and microcephaly. BMC Med Genomics. (2015) 9:7. 10.1186/s12920-016-0167-8 26846091 PMC4743197

[69] Duerinckx S Jacquemin V Drunat S Vial Y Passemard S Perazzolo C. Digenic inheritance of human primary microcephaly delineates centrosomal and non centrosomal pathways. Hum Mutat. (2019) 41:512–24. 10.1002/humu.23948 31696992 PMC7496698

[70] Noctor SC Martinez-Cerdeno V Ivic L Kriegstein AR. Cortical neurons arise in symmetric and asymmetric division zones and migrate through specific phases. Nat Neurosci. (2004) 7:136–44. 10.1038/nn1172 14703572

[71] Chenn A McConnell SK. Cleavage orientation and the asymmetric inheritance of Notch1 immunoreactivity in mammalian neurogenesis. Cell. (1995) 82:631–41. 10.1016/0092-8674(95)90035-7 7664342

[72] Noctor SC Flint AC Weissman TA Wong WS Clinton BK Kriegstein AR. Dividing precursor cells of the embryonic cortical ventricular zone have morphological and molecular characteristics of radial glia. J Neurosci. (2002) 22:3161–73. 10.1523/JNEUROSCI.22-08-03161.2002 11943818 PMC6757532

[73] Takahashi T Nowakowski RS Caviness VSJ. The leaving or Q fraction of the murine cerebral proliferative epithelium: a general model of neocortical neuronogenesis. J Neurosci. (1996) 16:6183–96. 10.1523/JNEUROSCI.16-19-06183.1996 8815900 PMC6579174

[74] Pucci B Kasten M Giordano A. Cell cycle and apoptosis. Neoplasia. (2000) 2:291–9. 10.1038/sj.neo.7900101 11005563 PMC1550296

[75] Sir J-H Barr AR Nicholas AK Carvalho OP Khurshid M Sossick A. A primary microcephaly protein complex forms a ring around parental centrioles. Nat Genet. (2011) 43:1147–53. 10.1038/ng.971 21983783 PMC3299569

[76] Kim T-S Park J-E Shukla A Choi S Murugan RN Lee JH. Hierarchical recruitment of Plk4 and regulation of centriole biogenesis by two centrosomal scaffolds, Cep192 and Cep152. Proc Natl Acad Sci USA. (2013) 110:4849–57. 10.1073/pnas.1319656110 24277814 PMC3864335

[77] Sonnen KF Gabryjonczyk AM Anselm E Nigg EA Stierhof YD. Human cep192 and cep152 cooperate in plk4 recruitment and centriole duplication. J Cell Sci. (2013) 126:3223–33. 10.1242/jcs.129502 23641073

[78] Tsuchiya Y Yoshiba S Gupta A Watanabe K Kitagawa D. Cep295 is a conserved scaffold protein required for generation of a bona fide mother centriole. Nat Commun. (2016) 7:12567. 10.1038/ncomms12567 27562453 PMC5007451

[79] Jayaraman D Kodani A Gonzalez DM Mancias JD Mochida GH Vagnoni C. Microcephaly proteins Wdr62 and Aspm define a mother centriole complex regulating centriole biogenesis, apical complex, and cell fate. Neuron. (2016) 92:813–28. 10.1016/j.neuron.2016.09.056 27974163 PMC5199216

[80] Pelletier L O'Toole E Schwager A Hyman AA Müller-Reichert T. Centriole assembly in *Caenorhabditis elegans*. Nature. (2006) 444:619–23. 10.1038/nature05318 17136092

[81] Keller D Orpinell M Olivier N Wachsmuth M Mahen R Wyss R. Mechanisms of HsSAS-6 assembly promoting centriole formation in human cells. J Cell Biol. (2014) 204:697–712. 10.1083/jcb.201307049 24590172 PMC3941056

[82] Rogala KB Dynes NJ Hatzopoulos GN Yan J Pong SK Robinson CV. The *Caenorhabditis elegans* protein SAS-5 forms large oligomeric assemblies critical for centriole formation. Elife. (2015) 4:1–51. 10.7554/eLife.07410 26023830 PMC4471805

[83] Delattre M Canard C Gönczy P. Sequential protein recruitment in *C. elegans* centriole formation. Curr Biol. (2006) 16:1844–9. 10.1016/j.cub.2006.07.059 16979563

[84] Singla V Romaguera-Ros M Garcia-Verdugo JM Reiter JF. Ofd1, a human disease gene, regulates the length and distal structure of centrioles. Dev Cell. (2010) 18:410–24. 10.1016/j.devcel.2009.12.022 20230748 PMC2841064

[85] Azimzadeh J Hergert P Delouvée A Euteneuer U Formstecher E Khodjakov A. hPOC5 is a centrin-binding protein required for assembly of full-length centrioles. J Cell Biol. (2009) 185:101–14. 10.1083/jcb.200808082 19349582 PMC2700515

[86] Schmidt TI Kleylein-Sohn J Westendorf J Le Clech M Lavoie SB Stierhof Y-D. Control of centriole length by CPAP and CP110. Curr Biol. (2009) 19:1005–11. 10.1016/j.cub.2009.05.016 19481458

[87] Lin YC Chang CW Hsu WB Tang CJC Lin YN Chou EJ. Human microcephaly protein CEP135 binds to hSAS-6 and CPAP, and is required for centriole assembly. EMBO J. (2013) 32:1141–4. 10.1038/emboj.2013.56 23511974 PMC3630357

[88] Dictenberg JB Zimmerman W Sparks CA Young A Vidair C Zheng Y. Pericentrin and gamma-tubulin form a protein complex and are organized into a novel lattice at the centrosome. J Cell Biol. (1998) 141:163–74. 10.1083/jcb.141.1.163 9531556 PMC2132723

[89] Hamill DR Severson AF Carter JC Bowerman B. Centrosome maturation and mitotic spindle assembly in *C. elegans* require SPD-5, a protein with multiple coiled-coil domains. Dev Cell. (2002) 3:673–84. 10.1016/S1534-5807(02)00327-1 12431374

[90] Haren L Stearns T Lüders J. Plk1-dependent recruitment of gamma-tubulin complexes to mitotic centrosomes involves multiple PCM components. PLoS ONE. (2009) 4:e5976. 10.1371/journal.pone.0005976 19543530 PMC2695007

[91] Doxsey SJ Stein P Evans L Calarco PD Kirschner M. Pericentrin, a highly conserved centrosome protein involved in microtubule organization. Cell. (1994) 76:639–50. 10.1016/0092-8674(94)90504-5 8124707

[92] Zheng Y Wong ML Alberts B Mitchison T. Nucleation of microtubule assembly by a γ-tubulin-containing ring complex. Nature. (1995) 378:578–83. 10.1038/378578a0 8524390

[93] Miyamoto T Akutsu SN Fukumitsu A Morino H Masatsuna Y Hosoba K. PLK1-mediated phosphorylation of WDR62/MCPH2 ensures proper mitotic spindle orientation. Hum Mol Genet. (2017) 26:4429–40. 10.1093/hmg/ddx330 28973348

[94] Seo MY Jang W Rhee K. Integrity of the pericentriolar material is essential for maintaining centriole association during m phase. PLoS ONE. (2015) 10:e138905. 10.1371/journal.pone.0138905 26407333 PMC4583256

[95] Cabral G Sans SS Cowan CR Dammermann A. Multiple mechanisms contribute to centriole separation in *C. elegans*. Curr Biol. (2013) 23:1380–7. 10.1016/j.cub.2013.06.043 23885867 PMC3722485

[96] Schlaitz A-L Srayko M Dammermann A Quintin S Wielsch N MacLeod I. The *C. elegans* RSA complex localizes protein phosphatase 2A to centrosomes and regulates mitotic spindle assembly. Cell. (2007) 128:115–27. 10.1016/j.cell.2006.10.050 17218259 PMC2987564

[97] Enos SJ Dressler M Gomes BF Hyman AA Woodruff JB. Phosphatase PP2A and microtubule-mediated pulling forces disassemble centrosomes during mitotic exit. Biol Open. (2018) 7:bio029777. 10.1101/182618 29222174 PMC5829501

[98] Tsou MFB Stearns T. Mechanism limiting centrosome duplication to once per cell cycle. Nature. (2006) 442:947–51. 10.1038/nature04985 16862117

[99] Fong K-W Choi Y-K Rattner JB Qi RZ. CDK5RAP2 is a pericentriolar protein that functions in centrosomal attachment of the gamma-tubulin ring complex. Mol Biol Cell. (2008) 19:115–25. 10.1091/mbc.e07-04-0371 17959831 PMC2174194

[100] Barrera JA Kao LR Hammer RE Seemann J Fuchs JL Megraw TL. CDK5RAP2 regulates centriole engagement and cohesion in mice. Dev Cell. (2010) 18:913–26. 10.1016/j.devcel.2010.05.017 20627074 PMC3078807

[101] Paramasivam M Yoon JC LoTurco JJ. ASPM and citron kinase co-localize to the midbody ring during cytokinesis. Cell Cycle. (2007) 6:1605–12. 10.4161/cc.6.13.4356 17534152

[102] Gruneberg U Neef R Li X Chan EHY Chalamalasetty RB Nigg EA. KIF14 and citron kinase act together to promote efficient cytokinesis. J Cell Biol. (2006) 172:363–72. 10.1083/jcb.200511061 16431929 PMC2063646

[103] Bassi ZI Audusseau M Riparbelli MG Callaini G D'Avino PP. Citron kinase controls a molecular network required for midbody formation in cytokinesis. Proc Natl Acad Sci USA. (2013) 110:9782–7. 10.1073/pnas.1301328110 23716662 PMC3683733

[104] Arora K Talje L Asenjo AB Andersen P Atchia K Joshi M. KIF14 binds tightly to microtubules and adopts a rigor-like conformation. J Mol Biol. (2014) 426:2997–3015. 10.1016/j.jmb.2014.05.030 24949858

[105] D'Avino PP Savoian MS Glover DM. Mutations in sticky lead to defective organization of the contractile ring during cytokinesis and are enhanced by Rho and suppressed by Rac. J Cell Biol. (2004) 166:61–71. 10.1083/jcb.200402157 15240570 PMC2172139

[106] Waddle JA Cooper JA Waterston RH. Transient localized accumulation of actin in *Caenorhabditis elegans* blastomeres with oriented asymmetric divisions. Development. (1994) 120:2317–28. 7925032 10.1242/dev.120.8.2317

[107] Nguyen-Ngoc T Afshar K Gönczy P. Coupling of cortical dynein and Gα proteins mediates spindle positioning in *Caenorhabditis elegans*. Nat Cell Biol. (2007) 9:1294–302. 10.1038/ncb1649 17922003

[108] Busson S Dujardin D Moreau A Dompierre J De Mey JR. Dynein and dynactin are localized to astral microtubules and at cortical sites in mitotic epithelial cells. Curr Biol. (1998) 8:541–4. 10.1016/S0960-9822(98)70208-8 9560347

[109] van der Voet M Berends CWH Perreault A Nguyen-Ngoc T Gönczy P Vidal M. NuMA-related LIN-5, ASPM-1, calmodulin and dynein promote meiotic spindle rotation independently of cortical LIN-5/GPR/Gα. Nat Cell Biol. (2009) 11:269–77. 10.1038/ncb1834 19219036

[110] Haren L Gnadt N Wright M Merdes A. NuMA is required for proper spindle assembly and chromosome alignment in prometaphase. BMC Res Notes. (2009) 2:64. 10.1186/1756-0500-2-64 19400937 PMC2686716

[111] Okumura M Natsume T Kanemaki MT Kiyomitsu T. Dynein–dynactin–NuMA clusters generate cortical spindle-pulling forces as a multiarm ensemble. Elife. (2018) 7:e36559. 10.7554/eLife.36559 29848445 PMC6037482

[112] Lorson MA Horvitz HR Van Den Heuvel S. LIN-5 is a novel component of the spindle apparatus required for chromosome segregation and cleavage plane specification in *Caenorhabditis elegans*. J Cell Biol. (2000) 148:73–86. 10.1083/jcb.148.1.73 10629219 PMC3207147

[113] Yao X Abrieu A Zheng Y Sullivan KF Cleveland DW. CENP-E forms a link between attachment of spindle microtubules to kinetochores and the mitotic checkpoint. Nat Cell Biol. (2000) 2:484–91. 10.1038/35019518 10934468

[114] Mao Y Desai A Cleveland DW. Microtubule capture by CENP-E silences BubR1-dependent mitotic checkpoint signaling. J Cell Biol. (2005) 170:873–80. 10.1083/jcb.200505040 16144904 PMC2171436

[115] Cheeseman IM Chappie JS Wilson-Kubalek EM Desai A. The conserved KMN network constitutes the core microtubule-binding site of the kinetochore. Cell. (2006) 127:983–97. 10.1016/j.cell.2006.09.039 17129783

[116] Sudakin V Chan GKT Yen TJ. Checkpoint inhibition of the APC/C in HeLa cells is mediated by a complex of BUBR1, BUB3, CDC20, and MAD2. J Cell Biol. (2001) 154:925–36. 10.1083/jcb.200102093 11535616 PMC2196190

[117] Kiyomitsu T Obuse C Yanagida M. Human Blinkin/AF15q14 is required for chromosome alignment and the mitotic checkpoint through direct interaction with Bub1 and BubR1. Dev Cell. (2007) 13:663–76. 10.1016/j.devcel.2007.09.005 17981135

[118] Bentley Lawrence J Villnave CA Singer RH. Sensitive, high-resolution chromatin and chromosome mapping *in situ*: presence and orientation of two closely integrated copies of EBV in a lymphoma line. Cell. (1988) 52:51–61. 10.1016/0092-8674(88)90530-2 2830981

[119] Li G Sudlow G Belmont AS Li G Willhelm C Sudlow G. Interphase cell cycle dynamics of a late-replicating, heterochromatic homogeneously staining region: precise choreography of condensation/decondensation and nuclear positioning. J Cell Biol. (1998) 140:975–89. 10.1083/jcb.140.5.975 9490713 PMC2132695

[120] Green LC Kalitsis P Chang TM Cipetic M Kim JH Marshall O. Contrasting roles of condensin I and condensin II in mitotic chromosome formation. J Cell Sci. (2012) 125:1591–604. 10.1242/jcs.097790 22344259 PMC3336382

[121] Yamashita D Shintomi K Ono T Gavvovidis I Schindler D Neitzel H. MCPH1 regulates chromosome condensation and shaping as a composite modulator of condensin II. J Cell Biol. (2011) 194:841–54. 10.1083/jcb.201106141 21911480 PMC3207293

[122] Rana V Bosco G. Condensin regulation of genome architecture. J Cell Physiol. (2017) 232:1617–25. 10.1002/jcp.25702 27888504

[123] Uhlmann F Lottspeich F Nasmyth K. Sister-chromatid separation at anaphase onset is promoted by cleavage of the cohesin subunit Scc1. Nature. (1999) 400:37–42. 10.1038/21831 10403247

[124] Chaplet M Rai R Jackson-Bernitsas D Li K Lin SY. BRIT1/MCPH1: a guardian of genome and an enemy of tumors. Cell Cycle. (2006) 5:2579–83. 10.4161/cc.5.22.3471 17172830

[125] Lin SY Rai R Li K Xu ZX Elledge SJ. BRIT1/MCPH1 is a DNA damage responsive protein that regulates the Brca1-Chk1 pathway, implicating checkpoint dysfunction in microcephaly. Proc Natl Acad Sci USA. (2005) 102:15105–9. 10.1073/pnas.0507722102 16217032 PMC1257745

[126] Peng G Yim EK Dai H Jackson AP van der Burgt I Pan MR. BRIT1/MCPH1 links chromatin remodelling to DNA damage response. Nat Cell Biol. (2009) 11:865–72. 10.1038/ncb1895 19525936 PMC2714531

[127] Holzer G Antonin W. Breaking the Y. PLoS Genet. (2019) 15:e1008109. 10.1371/journal.pgen.1008109 31120884 PMC6532837

[128] Asencio C Davidson IF Santarella-Mellwig R Ly-Hartig TBN Mall M Wallenfang MR. Coordination of kinase and phosphatase activities by Lem4 enables nuclear envelope reassembly during mitosis. Cell. (2012) 150:122–35. 10.1016/j.cell.2012.04.043 22770216

[129] Beukelaers P Vandenbosch R Caron N Nguyen L Belachew S Moonen G. Cdk6-dependent regulation of g1 length controls adult neurogenesis. Stem Cells. (2011) 29:713–24. 10.1002/stem.616 21319271

[130] Chen ZY Istfan NW. Docosahexaenoic acid, a major constituent of fish oil diets, prevents activation of cyclin-dependent kinases and S-phase entry by serum stimulation in HT-29 cells. Prostaglandins Leukot Essent Fat Acids. (2001) 64:67–73. 10.1054/plef.2000.0239 11161587

[131] Insua MF Garelli A Rotstein NP German OL Arias A Politi LE. Cell cycle regulation in retinal progenitors by glia-derived neurotrophic factor and docosahexaenoic acid. Investig Ophthalmol Vis Sci. (2003) 44:2235–44. 10.1167/iovs.02-0952 12714666

[132] Wang Y Chai Z Wang M Jin Y Yang A Li M. COPB2 suppresses cell proliferation and induces cell cycle arrest in human colon cancer by regulating cell cycle-related proteins. Exp Ther Med. (2018) 15:777–84. 10.3892/etm.2017.5506 29399086 PMC5772868

[133] Chan JP Wong BH Chin CF Galam DLA Foo JC Wong LC. The lysolipid transporter Mfsd2a regulates lipogenesis in the developing brain. PLoS Biol. (2018) 16:e2006443. 10.1371/journal.pbio.2006443 30074985 PMC6093704

[134] Nguyen LN Ma D Shui G Wong P Cazenave-Gassiot A Zhang X. Mfsd2a is a transporter for the essential omega-3 fatty acid docosahexaenoic acid. Nature. (2014) 509:503–6. 10.1038/nature13241 24828044

[135] Harel T Quek DQY Wong BH Cazenave-Gassiot A Wenk MR Fan H. Homozygous mutation in MFSD2A, encoding a lysolipid transporter for docosahexanoic acid, is associated with microcephaly and hypomyelination. Neurogenetics. (2018) 19:227–35. 10.1007/s10048-018-0556-6 30043326

[136] Kawakita E Hashimoto M Shido O. Docosahexaenoic acid promotes neurogenesis *in vitro* and *in vivo*. Neuroscience. (2006) 139:991–7. 10.1016/j.neuroscience.2006.01.021 16527422

[137] Bengoa-Vergniory N Gorroño-Etxebarria I González-Salazar I Kypta RM. A switch from canonical to noncanonical Wnt signaling mediates early differentiation of human neural stem cells. Stem Cells. (2014) 32:3196–208. 10.1002/stem.1807 25100239

[138] Chenn A Walsh CA. Regulation of cerebral cortical size by control of cell cycle exit in neural precursors. Science. (2002) 297:365–9. 10.1126/science.1074192 12130776

[139] Wexler EM Paucer A Kornblum HI Plamer TD Geschwind DH. Endogenous Wnt signaling maintains neural progenitor cell potency. Stem Cells. (2009) 27:1130–41. 10.1002/stem.36 19418460 PMC2782960

[140] Nicoleau C Varela C Bonnefond C Maury Y Bugi A Aubry L. Embryonic stem cells neural differentiation qualifies the role of Wnt/β-Catenin signals in human telencephalic specification and regionalization. Stem Cells. (2013) 31:1763–74. 10.1002/stem.1462 23818270

[141] Major MB Roberts BS Berndt JD Marine S Anastas J Chung N. New regulators of Wnt/β-catenin signaling revealed by integrative molecular screening. Sci Signal. (2008) 1:ra12. 10.1126/scisignal.2000037 19001663

[142] Buchman JJ Durak O Tsai LH. ASPM regulates Wnt signaling pathway activity in the developing brain. Genes Dev. (2011) 25:1909–14. 10.1101/gad.16830211 21937711 PMC3185963

[143] Pai VC Hsu CC Chan TS Liao WY Chuu CP Chen WY. ASPM promotes prostate cancer stemness and progression by augmenting Wnt–Dvl-3–β-catenin signaling. Oncogene. (2019) 38:1340–53. 10.1038/s41388-018-0497-4 30266990

[144] Rodríguez C Sánchez-Morán I Álvarez S Tirado P Fernández-Mayoralas DM Calleja-Pérez B. A novel human Cdh1 mutation impairs anaphase promoting complex/cyclosome activity resulting in microcephaly, psychomotor retardation, and epilepsy. J Neurochem. (2019) 151:103–15. 10.1111/jnc.14828 31318984 PMC6851713

[145] Maver A Cuturilo G Kovanda A Miletić A Peterlin B. Rare missense TUBGCP5 gene variant in a patient with primary microcephaly. Eur J Med Genet. (2019) 62:103598. 10.1016/j.ejmg.2018.12.003 30543990

[146] Richard EM Polla DL Assir MZ Contreras M Shahzad M Khan AA. Bi-allelic variants in METTL5 cause autosomal-recessive intellectual disability and microcephaly. Am J Hum Genet. (2019) 105:869–78. 10.1016/j.ajhg.2019.09.007 31564433 PMC6817559

[147] Chen T Zhang B Ziegenhals T Prusty AB Fröhler S Grimm C. A missense mutation in SNRPE linked to non-syndromal microcephaly interferes with U snRNP assembly and pre-mRNA splicing. PLoS Genet. (2019) 15:e1008460. 10.1371/journal.pgen.1008460 31671093 PMC6850558

[148] Martin CA Ahmad I Klingseisen A Hussain MS Bicknell LS Leitch A. Mutations in PLK4, encoding a master regulator of centriole biogenesis, cause microcephaly, growth failure and retinopathy. Nat Genet. (2014) 46:1283–92. 10.1038/ng.3122 25344692 PMC4676084

[149] Shohayeb B Ho U Yeap YY Parton RG Millard SS Xu Z. The association of microcephaly protein WDR62 with CPAP/IFT88 is required for cilia formation and neocortical development. Hum Mol Genet. (2020) 29:248–63. 10.1093/hmg/ddz281 31816041

[150] Simmons AJ Park R Sterling NA Jang MH Van Deursen JMA Yen TJ. Nearly complete deletion of BubR1 causes microcephaly through shortened mitosis and massive cell death. Hum Mol Genet. (2019) 28:1822–36. 10.1093/hmg/ddz022 30668728 PMC6522074

[151] Ding W Wu Q Sun L Pan NC Wang X. Cenpj regulates cilia disassembly and neurogenesis in the developing mouse cortex. J Neurosci. (2019) 39:1994–2010. 10.1523/JNEUROSCI.1849-18.2018 30626697 PMC6507096

[152] Blachon S Gopalakrishnan J Omori Y Polyanovsky A Church A Nicastro D. Drosophila asterless and vertebrate Cep152 are orthologs essential for centriole duplication. Genetics. (2008) 180:2081–94. 10.1534/genetics.108.095141 18854586 PMC2600943

[153] Zheng X Gooi LM Wason A Gabriel E Mehrjardi NZ Yang Q. Conserved TCP domain of Sas-4/CPAP is essential for pericentriolar material tethering during centrosome biogenesis. Proc Natl Acad Sci USA. (2014) 111:354–63. 10.1073/pnas.1317535111 24385583 PMC3903230

[154] Singh P Ramdas Nair A Cabernard C. The centriolar protein Bld10/Cep135 is required to establish centrosome asymmetry in drosophila neuroblasts. Curr Biol. (2014) 24:1548–55. 10.1016/j.cub.2014.05.050 24954048

[155] Ogungbenro YA Tena TC Gaboriau D Lalor P Dockery P Philipp M. Centrobin controls primary ciliogenesis in vertebrates. J Cell Biol. (2018) 217:1205–15. 10.1083/jcb.201706095 29440264 PMC5881496

[156] Gao XL Tian WJ Liu B Wu J Xie W Shen Q. High-mobility group nucleosomal binding domain 2 protects against microcephaly by maintaining global chromatin accessibility during corticogenesis. J Biol Chem. (2020) 295:468–80. 10.1074/jbc.RA119.010616 31699896 PMC6956539

[157] Pelletier L Özlü N Hannak E Cowan C Habermann B Ruer M. The *Caenorhabditis elegans* centrosomal protein SPD-2 is required for both pericentriolar material recruitment and centriole duplication. Curr Biol. (2004) 14:863–73. 10.1016/j.cub.2004.04.012 15186742

[158] Liao C Fu F Li R Yang WQ Liao HY Yan JR. Loss-of-function variation in the DPP6 gene is associated with autosomal dominant microcephaly and mental retardation. Eur J Med Genet. (2013) 56:484–9. 10.1016/j.ejmg.2013.06.008 23832105

[159] Yigit G Brown KE Kayserili H Pohl E Caliebe A Zahnleiter D. Mutations in cdk5rap2 cause seckel syndrome. Mol Genet Genomic Med. (2015) 3:467–80. 10.1002/mgg3.158 26436113 PMC4585455

[160] Darvish H Esmaeeli-Nieh S Monajemi GB Mohseni M Ghasemi-Firouzabadi S Abedini SS. A clinical and molecular genetic study of 112 Iranian families with primary microcephaly. J Med Genet. (2010) 47:823–8. 10.1136/jmg.2009.076398 20978018

[161] Shi L Li M Lin Q Qi X Su B. Functional divergence of the brain-size regulating gene MCPH1 during primate evolution and the origin of humans. BMC Biol. (2013) 11:62. 10.1186/1741-7007-11-62 23697381 PMC3674976

[162] Zhang J. Evolution of the human ASPM gene, a major determinant of brain size. Genetics. (2003) 165:2063–70. 14704186 10.1093/genetics/165.4.2063 PMC1462882

[163] Montgomery SH Capellini I Venditti C Barton RA Mundy NI. Adaptive evolution of four microcephaly genes and the evolution of brain size in anthropoid primates. Mol Biol Evol. (2011) 28:625–38. 10.1093/molbev/msq237 20961963

[164] Evans PD Vallender EJ Lahn BT. Molecular evolution of the brain size regulator genes CDK5RAP2 and CENPJ. Gene. (2006) 375:75–9. 10.1016/j.gene.2006.02.019 16631324

[165] Fiddes IT Lodewijk GA Mooring M Bosworth CM Ewing AD Mantalas GL. Human-specific NOTCH2NL genes affect notch signaling and cortical neurogenesis. Cell. (2018) 173:1356–69. 10.1016/j.cell.2018.03.051 29856954 PMC5986104

[166] Gilbert SL Dobyns WB Lahn BT. Genetic links between brain development and brain evolution. Nat Rev Genet. (2005) 6:581–90. 10.1038/nrg1634 15951746

[167] Koreth J van den Heuvel S. Cell-cycle control in *Caenorhabditis elegans*: how the worm moves from G1 to S. Oncogene. (2005) 24:2756–64. 10.1038/sj.onc.1208607 15838512

[168] Calegari F Haubensak W Haffher C Huttner WB. Selective lengthening of the cell cycle in the neurogenic subpopulation of neural progenitor cells during mouse brain development. J Neurosci. (2005) 25:6533–8. 10.1523/JNEUROSCI.0778-05.2005 16014714 PMC6725437

[169] Yeo G Holste D Kreiman G Burge CB. Variation in alternative splicing across human tissues. Genome Biol. (2004) 5:R74. 10.1186/gb-2004-5-10-r74 15461793 PMC545594

[170] Omer Javed A Li Y Muffat J Su KC Cohen MA Lungjangwa T. Microcephaly modeling of kinetochore mutation reveals a brain-specific phenotype. Cell Rep. (2018) 25:368–82. 10.1016/j.celrep.2018.09.032 30304678 PMC6392048

[171] Shaheen R Maddirevula S Ewida N Alsahli S Abdel-Salam GMH Zaki MS. Genomic and phenotypic delineation of congenital microcephaly. Genet Med. (2019) 21:545–52. 10.1038/s41436-018-0140-3 30214071 PMC6986385

